# Analytical Models to Optimize Tacrolimus Dosing in Solid Organ Transplantation: A Systematic Review

**DOI:** 10.3390/pharmaceutics18040430

**Published:** 2026-03-31

**Authors:** Elmira Amooei, Nandini Biyani, Amos Buh, Martin M. Klamrowski, Nawaf M. Alyahya, Christopher R. McCudden, James R. Green, Babak Rashidi, Haya Almuzirai, Stephanie Hoar, Ayub Akbari, Gregory L. Hundemer, Ran Klein

**Affiliations:** 1 Department of Systems and Computer Engineering, Carleton University, Ottawa, ON K1S 5B6, Canada; jrgreen@sce.carleton.ca; 2 Kidney Research Centre, Ottawa Hospital Research Institute, Ottawa, ON K1H 8L6, Canada; nbiyani@ohri.ca (N.B.); abuh020@uottawa.ca (A.B.); aakbari@toh.ca (A.A.); ghundemer@toh.ca (G.L.H.); 3 Faculty of Health Sciences, University of Ottawa, Ottawa, ON K1N 6N5, Canada; 4 Department of Mechanical Engineering, University of Ottawa, Ottawa, ON K1N 6N5, Canada; mklamrowski@ohri.ca; 5 Division of Nephrology, Department of Internal Medicine, College of Medicine, Imam Abdurahman Bin Faisal University, Al Khobar 31441, Saudi Arabia; nmalyahyah@iau.edu.sa; 6 Eastern Ontario Regional Laboratory Association, Ottawa, ON K1H 8L6, Canada; cmccudde@uottawa.ca; 7 Division of Biochemistry, Department of Pathology and Laboratory Medicine, University of Ottawa, Ottawa, ON K1N 6N5, Canada; 8 Division of General Internal Medicine, Department of Medicine, University of Ottawa, Ottawa, ON K1N 6N5, Canada; brashidi@toh.ca (B.R.); halmuzirai@toh.ca (H.A.); 9 Division of Nephrology, Department of Medicine, University of Ottawa, Ottawa, ON K1N 6N5, Canada; shoar@toh.ca; 10 Methodological and Implementation Research Program, Ottawa Hospital Research Institute, University of Ottawa, Ottawa, ON K1N 6N5, Canada; 11 Division of Nuclear Medicine, Department of Medicine, University of Ottawa, Ottawa, ON K1N 6N5, Canada

**Keywords:** tacrolimus, drug dose monitoring, dose optimization, organ transplantation

## Abstract

**Background:** Tacrolimus dose optimization remains challenging due to its narrow therapeutic range and multiple influencing variables. This systematic review aimed to identify effective analytical modeling techniques for optimal tacrolimus dose prediction in solid organ transplant recipients. **Methods:** Two independent researchers conducted a comprehensive review of studies examining analytical models that optimize tacrolimus dosing, searching Medline, Scopus, Embase, Web of Science, and PubMed. **Results:** In total, 115 studies met the inclusion criteria. Pharmacokinetic models (74 studies), particularly two-compartment with Bayesian forecasting, were most frequently used. Machine learning (ML) approaches, with increasing adoption, have demonstrated promising improved predictive accuracy. Key predictive variables included CYP3A5 genotype, hematocrit levels, post-operative days, and weight; however, the significance of genomic features seemed to diminish progressively as therapeutic drug monitoring calibrates dosing in the months following post-transplant. Only ten studies performed external validation, and none incorporated adherence data or predicted long-term graft outcomes. **Conclusions:** Clinical deployment of predictive models for tacrolimus dosing remains uncommon. In research, pharmacokinetic models remain prevalent, with ML approaches showing early incremental promise. Limited external validation raises generalizability concerns. Future research should prioritize outcome-based evaluation metrics rather than error metrics.

## 1. Introduction

Tacrolimus is a calcineurin inhibitor frequently used as an immunosuppressive agent in solid organ transplantation to prevent graft rejection for kidney, liver, pancreas, heart, and lung transplant recipients [1,2]. Tacrolimus has a narrow therapeutic window with serious complications from under- or overdosing [3]. Overdosing can induce significant nephro- and neurotoxicity [4,5], while underdosing may lead to organ rejection [4,6,7]. Optimal dosing is challenging due to complex pharmacokinetics and drug–drug and food–drug interactions [4,5].

Initial dosing is typically patient weight-based, followed by iterative adjustments informed by trough blood concentration measurements. Unfortunately, this approach often fails to reach target concentrations [8], with studies showing that only 37% of kidney transplant recipients achieve target ranges when following the traditional weight-based dosing [8,9]. Target ranges are evidence-based and vary by organ and post-transplant periods. Furthermore, patient responses vary substantially, due to factors including genetics (CYP3A4 and CYP3A5 polymorphism) [10,11,12], demographics [12,13,14], laboratory parameters (albumin, hematocrit, and liver function) [12,15], and various drugs (notably CYP3A inhibitors like fluconazole) [15] and food interactions (e.g., grapefruit) [16,17].

The variability of blood tacrolimus concentration is further exacerbated by complex pharmacokinetics. After oral administration, it is absorbed through the intestines at a rate varying by individual, distributed by binding to erythrocytes and plasma proteins, metabolized through CYP3A enzymes, and excreted mainly through biliary routes [18]. The key pharmacokinetic parameters are defined in Table 1.

While a single-compartment model uses pharmacokinetic parameters to predict future drug concentrations, tacrolimus behavior is often more complex [15]. To address these challenges, researchers have developed more sophisticated models, including multi-compartment models, Bayesian estimation, ML approaches, and statistical models [19,20,21,22]. These models integrate multiple compartments associated with tacrolimus metabolism, storage, and clearance to more accurately model concentrations at arbitrary time intervals [18,21,23,24].

Conversely, trough-level concentrations represent the most simplified tacrolimus pharmacokinetic model, evaluating blood concentrations immediately preceding the next dose (example at 12 or 24 h post dose depending on formulation). Trough levels are relatively easily measured in the outpatient setting. The patient response to tacrolimus can be succinctly summarized as the ratio of trough-level concentrations to prescribed dose (C/D) once sufficient dosing cycles have elapsed to reach a steady state (e.g., after at least 7 days since last dose change).

Despite numerous studies exploring predictive models for tacrolimus dosing, a comprehensive synthesis of modeling approaches is lacking [18,19,21,22,25,26,27,28,29,30]. This systematic review explores the literature on various endpoints, including concentration, trough, and dose prediction studies, beyond the pharmacokinetic modeling methods that are widely investigated. We include ML approaches and summarize the most significant predictors influencing tacrolimus blood concentrations, describe the importance of genomics, and synthesize existing evidence to guide the future development of predictive models as clinical dosing decision aids. Our review is complementary to other reviews, such as the work of Hoffert et al. (2024) [31] and Lloberas et al. (2025) [32], in that we identify a larger corpus of studies incorporating both popPK and ML approaches, and systematically explore endpoints, predictive covariates, and error structures.

## 2. Materials and Methods

This systematic review followed the Preferred Reporting Items for Systematic Reviews and Meta-Analyses (PRISMA, Supplementary Material S1) [33] guidelines and was registered with the International Prospective Register of Systematic Reviews (PROSPERO) [34] (CRD42024537212). The protocol was previously published [35].

### 2.1. Inclusion Criteria

We used PICO (Population, Intervention, Comparison, Outcome) criteria to select the right studies: **Population:** Adult patients (≥18 years) who underwent solid organ transplantation (e.g., kidney, liver) with analytical tacrolimus dose/concentration prediction models. **Intervention:** Analytical models or methods (statistical, ML, Bayesian, or kinetic) for tacrolimus dose prediction and/or maintenance of its blood concentration. **Comparison:** Alternative methods, such as clinician discretion. **Outcomes:** Significant covariates influencing tacrolimus concentration, and metrics used to evaluate model performance.

### 2.2. Types of Included Studies

Experimental study designs, including before-and-after studies, cross-sectional studies, cohort studies, qualitative studies, and randomized control trials (RCTs), in English or French, were included regardless of the publication year.

### 2.3. Search Strategy

Systematic searches were conducted in databases including Ovid/MEDLINE, PubMed/MEDLINE, Scopus, Web of Science, and Embase (1946–11 March 2024) in collaboration with a librarian (RS). Search terms included controlled terms and free-text terms: ‘tacrolimus’, ’dose prediction’, ‘machine learning’, ‘Bayesian theorem’, and ‘kinetic modeling’. Search was limited to human and adult studies. The full search strategy is in Supplementary Material S2. Grey literature was explored through Google Scholar. Conference abstracts published within two years of the search date (published in and after January 2022) were included.

### 2.4. Study Selection and Eligibility Criteria

Studies were imported into the Covidence software © 2024 [36] for screening. Duplicates were identified through Covidence or through manual screening. Two independent reviewers (EA, MMK) performed title–abstract and full-text screening; disagreements were resolved by a third reviewer (AB). Data extraction was performed using the Joanna Briggs Institute Meta-Analysis of Statistics Assessment and Review instrument [37] (Supplementary Material S3). Four reviewers (EA, NB, MMK, NA) extracted data; discrepancies were resolved by EA.

### 2.5. Quality of Evidence and Risk-of-Bias Assessment

Evidence quality was assessed using the Grading of Recommendations Assessment, Development and Evaluation (GRADE) [38]. Risk of bias was assessed using the Joanna Briggs Institute (JBI) Critical Appraisal Checklist for Systematic Reviews and Research Syntheses [37] by two independent reviewers (EA, NB). Disagreements were resolved through discussion.

### 2.6. Data Synthesis

A narrative synthesis was conducted. Only four studies had sufficient data for meta-analyses; due to this limitation, we refrained from conducting a meta-analysis.

## 3. Results

### 3.1. Study Characteristics

**Figure 1:**
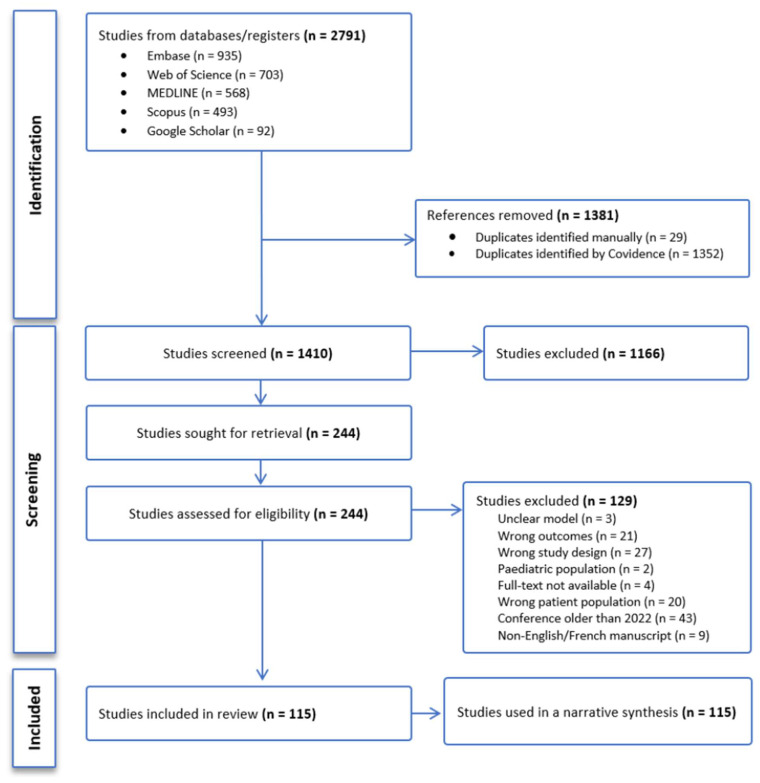
PRISMA flow diagram for study selection.

**Figure 2:**
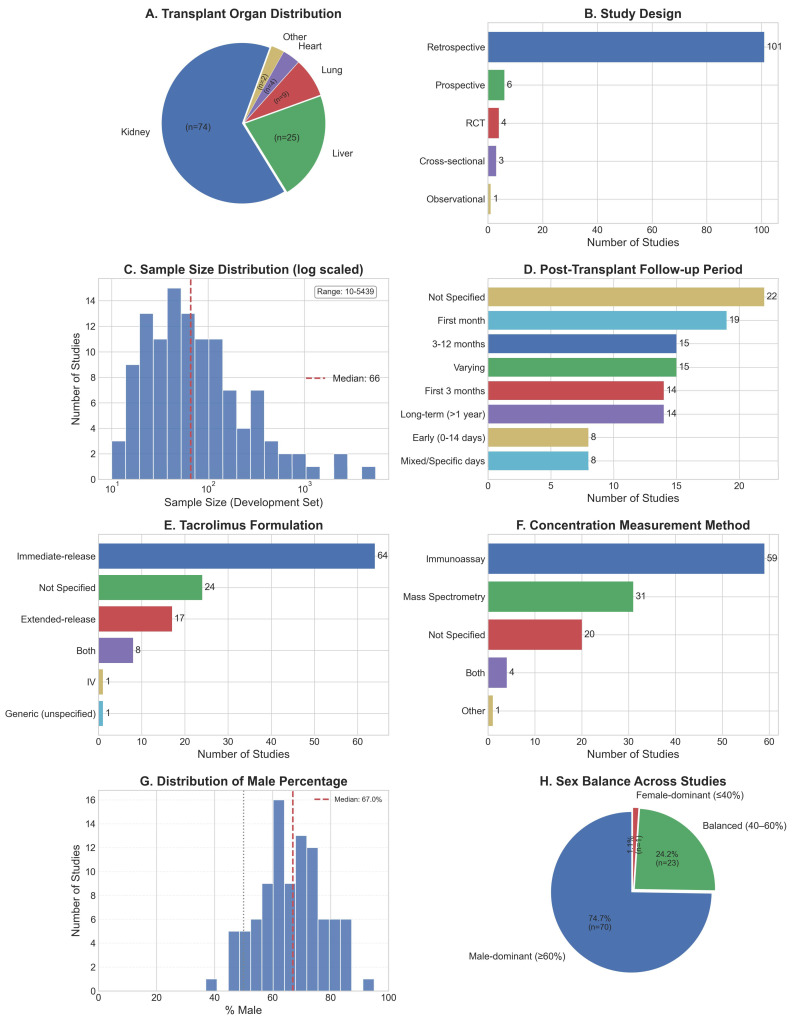
Characteristics of the included studies (*n* = 115). Panel (**A**) shows the distribution of the transplanted organs studied, with 74 (64%) exploring kidney transplant recipients as their primary population, and “other” referring to multiorgan studies. Panel (**B**) shows different study methodologies, with the vast majority (88%) being retrospective in design. Panel (**C**) is a histogram of study population sizes, which ranged from 10 to 5439 patients. Panel (**D**) summarizes the post-transplant follow-up period of patients in each study. Panel (**E**) summarizes the tacrolimus formulations studied, with immediate release being the most common (56%). Panel (**F**) summarizes the tacrolimus blood concentration measurement methods used in each study, with immunoassay techniques being the most common (51%). Panel (**G**) is a histogram of the distribution of male percentage population across studies, with a median of 67% male population for model development. Panel (**H**) summarizes the number of studies with over 60% male population (*n* = 70) and one study with below a 40% male population.

A total of 115 studies were included for narrative synthesis (Table 2, Figure 1). The predominant focus was on kidney (74 studies) and liver (25 studies) transplant recipients (Figure 2A). Most studies were retrospective in design (101 studies), with only six prospective and four clinical trials (Figure 2B).

**Figure 3:**
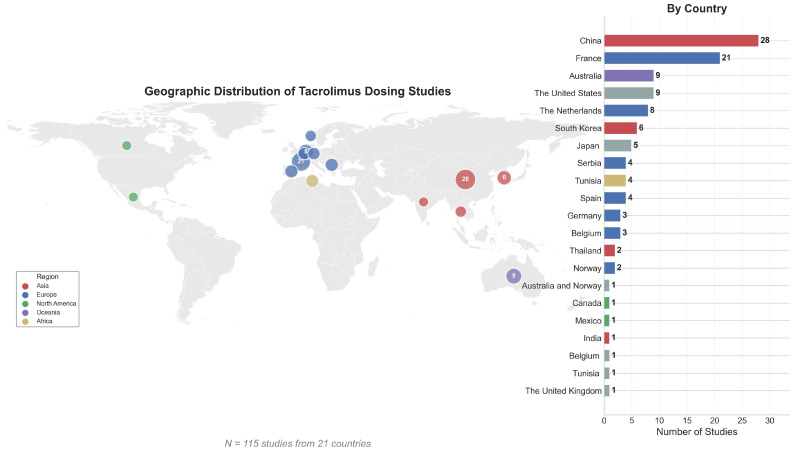
Frequency of study countries and their geographic distributions included in the review (*n* = 115).

Patient cohort sizes varied widely (10–5439 with a median of 66 patients, Figure 2C); as did post-operative follow-up periods, spanning from early post-operative days (0–14 days in 8 studies) to long-term (>1 year in 14 studies), with 41 studies focusing on the first 3 months of the post-transplant period (Figure 2D). Sixty-four studies investigated the immediate-release formulation (Prograf^®^), followed by the extended-release (Advagraf^®^, 17 studies) (Figure 2E). Tacrolimus trough whole-blood concentrations were most commonly measured using immunoassays (59 studies, Figure 2F) or mass spectrometry (31 studies). The median male population for model development was 67% (Figure 2G), and 70 studies (75%) had a male-dominant cohort (i.e., ≥60% male population, Figure 2H). The largest number of studies originated from China (28), followed by France (21 studies) (Figure 3).

### 3.2. Quality Appraisal of Studies

Following JBI critical appraisal guidelines, 99 papers (86%) were scored as a high risk of bias (>60%), mainly due to a failure to provide a clear description of dataset split or patient censoring methods. Moreover, 64 studies (56%) failed to explicitly state the inclusion criteria; however, all studies specified the participants’ characteristics (Supplementary Material S3).

### 3.3. Prediction Targets

**Figure 4:**
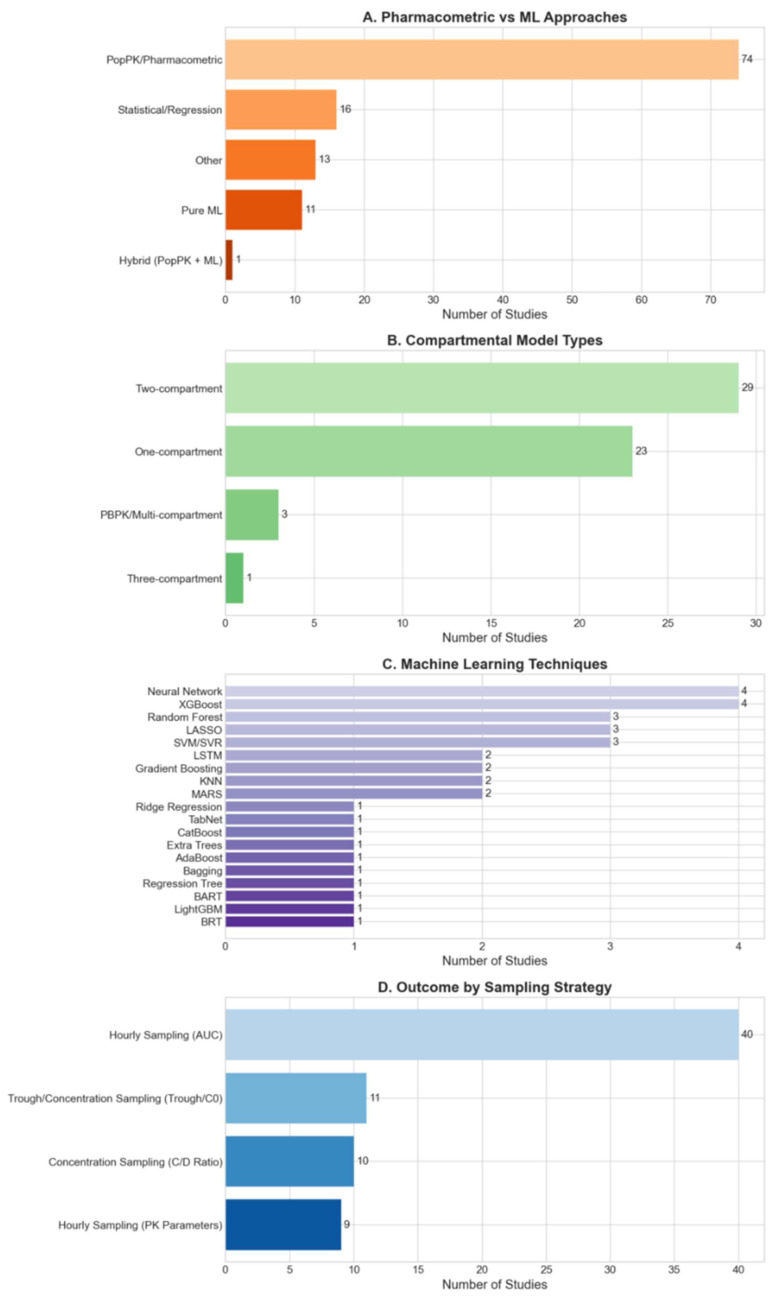
Distribution of different modeling techniques. Panel (**A**) compares overall modeling approaches in reviewed studies (*n* = 115), with the most common approach (*n* = 74, 64%) being popPK modeling. Panel (**B**) compares different compartmental models, with 29 studies utilizing two-compartment models, followed by 23 utilizing one-compartment models. Panel (**C**) shows various ML approaches (XGBoost = Extreme Gradient Boosting, LASSO = Least Absolute Shrinkage and Selection Operator, SVM = Support Vector Machine, SVR = Support Vector Regression, KNN = K-Nearest Neighbor, MARS = Multivariate Adaptive Regression Spline, TabNet = Tabular Network, CatBoost = Categorical Boosting, AdaBoost = Adaptive Boosting, BART = Bayesian Additive Regression Trees, LightGBM = Light Gradient Boosting Machine, BRT= Boosted Regression Trees) explored, with Neural Networks and XGBoost being the most common. Panel (**D**) shows predicted target based on sampling strategies where hourly sampling is used in AUC prediction studies and pharmacokinetic parameter predictions, and daily sampling is used to trough or concentration prediction studies.

Prediction targets varied amongst the studies, underscoring different clinical objectives for tacrolimus management. These were divided into concentration prediction (to illustrate hourly concentration changes post-dose, for long- and short-term management), trough prediction (to predict the lowest concentration in the blood before next dosing, for long- and short-term management), and dose recommendation. Trough prediction is fundamentally different from (hourly) concentration prediction, as trough prediction captures a single blood sample prediction before the next dose is administered. Concentration prediction, using area under the curve (AUC) calculations, measures the systemic drug exposure over an entire period between two dosing events, which requires multiple blood samples collected at specific timepoints (specific post-dose hours, Figure 4D).

In AUC modeling, the input data should be verified for an accurate dose and collection time, as a sample collection time mismatch could result in substantial shifts in AUC predictions. Trough prediction models are slightly more flexible regarding sampling time mismatch, but do not capture intra-dose variabilities, especially in inpatient and immediate post-transplant populations due to the tacrolimus half-life.

No studies predicted long-term (>1 year) outcomes like graft survival directly. Clinical thresholds for safe or unsafe predictions were explicitly incorporated in 44 studies, typically defining therapeutic blood concentration ranges (e.g., 8–12 ng/mL in early post-transplant, 5–10 ng/mL later).

### 3.4. Tacrolimus Concentration Prediction

Forty studies predicted 12 or 24 h average tacrolimus blood levels (i.e., between dosing) [13,25,27,28,29,47,52,53,59,60,64,70,75,80,88,97,98,99,102,106,111,113,115,116,117,118,119,120,121,125,126,127,129,130,132,138,139,140,141]. For AUC_12_ prediction, C_2_ (concentration at two hours post-dose) was most frequently concluded to be the optimal single-point surrogate (18 studies), followed by C_4_ (12 studies) [47,60,64,88,99,111,115,118,121,125,126,127,129,138]. Multi-point strategies (C_0_ + C_2_ + C_4_) improved accuracy in eight studies. For AUC_24_, C_2_ and C_2.5_ were strong predictors (10 studies), alongside C_0_ and C_3_ [53,70,97,102,116,121,125,141].

### 3.5. Tacrolimus Trough and Dose Prediction

The next-day trough prediction was modeled in 31 studies. Hybrid targets combining dose recommendation and response prediction were explored in 15 of these studies. Twenty–seven studies focused on the next dose (for different post-transplant periods) [10,12,14,18,21,29,43,55,56,65,66,71,74,81,90,94,96,101,105,108,112,113,134,135] or initial-dose [10,39,44,72,76,93,128] prediction, emphasizing short-term inpatient management.

### 3.6. Modeling Techniques

Population pharmacokinetic (popPK) models dominated (74 studies), especially using two-compartment models (29 studies) [12,13,18,22,27,39,41,42,44,45,52,55,92,115,118,121,122,124,130,135,141] followed by one-compartment modeling (23 studies, Figure 4B) [29,45,57,61,66,71,78,79,81,82,89,91,92,96,97,98,112,113,115,116,123,127,141,142]. Bayesian estimation was frequently paired with two-compartment popPK models (29 studies), using NONMEM^®^ (58 studies), or Pmetric (12 studies). Gérard et al. developed a 13-compartment physiologically based pharmacokinetic (PBPK) model [76], and Pei et al. used a 15-compartment model [110], representing the most complex structural approaches. All of these models processed longitudinal data over the entire available history, including demographics and tacrolimus concentration.

### 3.7. Post-Transplant Phase Modeling

Studies can be categorized into three modeling approached according to how post-transplant phases are managed: phase-specific models [45,113] that are designed for a specific time window, temporal covariate unified models [40,122] to capture post-transplant trajectory, and stable-only models [12,102] that deliberately exclude early stages. None of the three categories are restricted to inpatient or outpatient settings. Tacrolimus CL/F undergoes substantial changes from immediate to stable post-operative periods due to hepatic regeneration and hematocrit recovery, which directly influences model selection.

The clinical utility of CYP3A5 genotyping is equally phase-dependent. High-dose corticosteroids early post-transplant pharmacologically increase CYP3A4 expression, masking genotypic differences [122]. Woillard et al. (2011) further observed that the dose-requirement advantage of CYP3A5 expressors disappears by 6–12 months once therapeutic drug monitoring has stabilized individual doses [130].

Collectively, these suggest that model selection and the relevance of specific covariates should therefore always be considered relative to the post-transplant phase for which a model was developed and validated.

**Figure 5:**
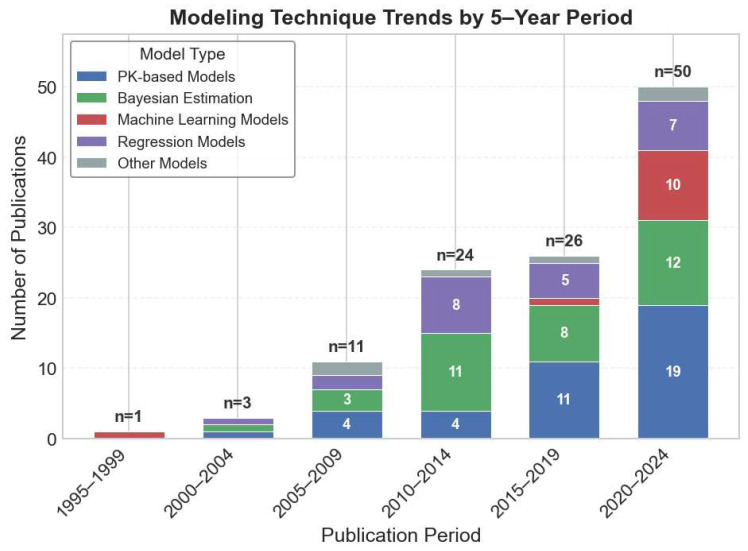
Trend of publications using different modeling techniques in recent years.

Figure 5 summarizes modeling techniques over the past 30 years. ML approaches to modeling tacrolimus levels were described as early as 1999 [58] but then fell dormant. Coinciding with the rise of ML in general, 10 of 50 studies (20%) published after 2020 used ML. XGBoost [21,74,91,128,132,134,135,143] and Artificial Neural Networks (ANNs) [21,58,74,135] were the most commonly (4 studies each) explored ML methods (Figure 4C).

### 3.8. Predictive Covariates

**Figure 6:**
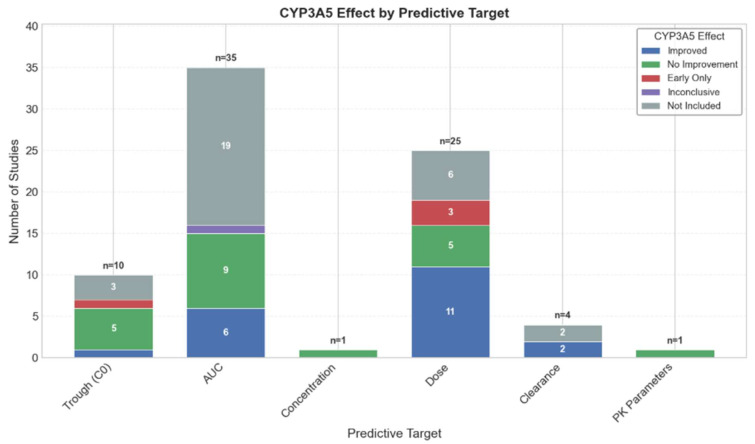
The significance of CYP3A5 in different studies by target predictions. This figure summarizes CYP3A5 inclusion as a predictive covariate for different endpoints.

Eighty-three studies explored significant predictors affecting tacrolimus. The CYP3A5 genotype appeared in 66 of these studies and ranked highest in covariate analyses, reflecting its central role in tacrolimus metabolism by CYP3A [18,51,54,64,67,74,84,107,122,144]. However, the significance of the CYP3A genotype varied by predictive target (Figure 6).

CYP3A5 expressers were consistently reported to require 1.2–2.2 times higher doses to achieve a target blood level. However, the clinical utility of CYP3A5 genotyping varied with the post-transplant timeframe. Of 52 studies examining CYP3A5 temporal significance, 22 studies found no significant benefit at any timepoint, 18 studies showed a sustained benefit beyond the first week post-transplant [39,40,43,50,54,78,81,84,89,96,102,107,119,135,137,144], while 12 reported diminishing predictive value after the initial post-transplant period [12,27,61,63,67,71,75,82,86,118,121,127]. Niioka et al. specifically demonstrated that CYP3A5 genotype utility was most pronounced after day 14 post-transplant [107]. Notably, Kirubakaran et al., 2023 [86], and Storset et al., 2022, found that once trough concentration history becomes available, phenotypical response data may supersede genomics for daily dose adjustments. In liver transplant recipients, the donor CYP3A5 genotype might be particularly important beyond the first 3 months as hepatic metabolism influences tacrolimus clearance [75,102].

For AUC prediction, recent trough levels and dose history became more predictive than genomics, suggesting that phenotypic response data encompass more pharmacokinetic information than the genotype alone.

Hematocrit (39 studies) consistently affected the clearance rate (CL/F) as tacrolimus mechanistically binds to red blood cells. Post-operative days (POD) followed (36 studies), representing the time-dependent recovery of hepatic enzyme activity. Weight (28 studies) consistently affected the volume of distribution (*V_d_*). Demographics, including age (21 studies) and sex (11 studies), were significant, representing age- and sex-related metabolic changes, followed by serum albumin and serum creatinine (27 studies combined), representing hepatic and kidney function. Co-medication including azole antifungals (20 studies) was highlighted as a significant covariate, consistent with their role as CYP3A inhibitors.

**Figure 7:**
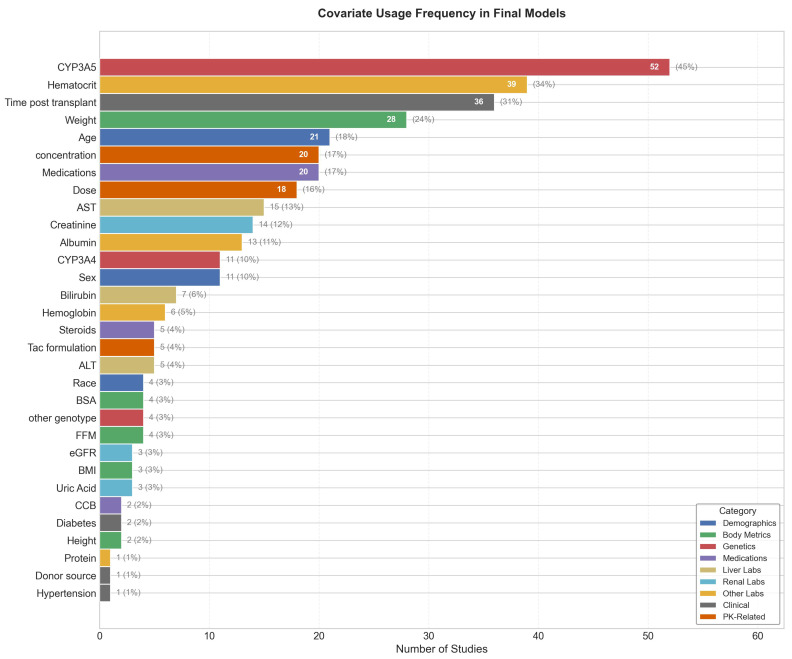
Frequency of different covariates and their categories used in final model development.

Figure 7 summarizes the frequency of studies that incorporated each covariate in their final model.

The CYP3A5 * 1 allele in kidney and liver studies is reported to be the most clinically important, with effects ranging from 26% to an over 3-fold increase in CL/F depending on the population (Supplementary Material S6). This large variability likely suggests that model estimates are sensitive towards population compositions, time post-transplant, and how CYPS3A5 genotypes are split. Reséndiz-Galván et al. reported a 30% vs. 39% hematocrit, the second most frequent covariate, to result in approximately 8–10% higher CL/F, which reflects a mechanistic tacrolimus behavior of having fewer red blood cells, resulting in faster free drug clearance.

### 3.9. Model Performance, Calibration, and Error Handling

#### 3.9.1. Performance Metrics

Performance metrics varied by modeling technique, with studies either assessing the performance in achieving target concentrations or evaluating the dosing accuracy in retrospective data. These metrics fell into three main categories: (1) bias/precision metrics indicating prediction error including median prediction error (MPE) and mean absolute prediction error (MAPE) (78 studies); (2) model fit metrics assessing correlation between predicted and observed values (R^2^, RMSE or root mean square error) (52 studies); and (3) clinical metrics (fraction within acceptable ranges (prediction errors within ±20% of the actual values or F20, and prediction errors within ±30% of the actual values or F30)) to evaluate target achievement and maintenance (29 studies). Complete definitions for these metrics are provided in Supplementary Material S4. Tables S10 and S11 in Supplementary Material S6 compare the error metrics between popPK and ML models for kidney and liver models.

#### 3.9.2. Model Calibration

Model calibration (agreement between observed and predicted probability) was assessed through visual predictive checks (VPCs) in 42 studies and goodness-of-fit plots (GOF) in 58 studies. Notably, calibration at extreme values (very high or very low concentrations) was rarely explicitly studied, revealing a potentially important negligence of extreme concentration scenarios, where clinical consequences could be most severe.

#### 3.9.3. Residual Error Handling

Residual errors between model-predicted and measured blood concentrations were reported in 92 studies. popPK models frequently (65 of 74 studies) reported residual errors. ML and regression methods (18 studies) relied on residual error-based regularization (L1/L2) to prevent overfitting (e.g., by tree pruning).

### 3.10. External Validation and Generalizability

Thirty-eight studies performed internal validation using bootstrapping. Only ten studies evaluated model performance on external data, showing limited transferability and generalizability within and across different organs, as these models performed poorly on external validation data [18,25,26,27,28,29,30,51,56].

The externally validated model development studies shared several distinguishing characteristics, including larger and more diverse development cohorts (e.g., Al-Kofahi et al. 2021: *n* = 608 development, *n* = 1361 external validation recipients), the CYP3A5 genotype and multi-covariate physiological frameworks within their covariate structures, and in some cases, prospective validation designs [51,71]. Their reported validation performance was, on balance, superior to internally validated models of a similar scale (Supplementary Material S6). However, standalone evaluation studies caution strongly against interpreting single-population external validation as evidence of broad generalizability. For instance, Methaneethorn et al., 2022 [101], found only 3 of 10 published models acceptable in a Thai kidney transplant cohort, and Kirubakaran et al., 2022 [19], found that all 17 evaluated models were systematically underpredicted in patients receiving concomitant azole antifungal therapy, regardless of the original validation status. Across external validation studies, prediction errors within ±30% of actual values (F30) were achieved in fewer than 50% of the predictions, with individual patient prediction errors ranging from 60% to ≥200% [18]. Zhao et al., 2016 [18], demonstrated that Bayesian priors can significantly boost performance in externally validated models, suggesting that incorporating prior population information may improve generalizability.

### 3.11. Interpretability

Interpretability, the extent to which humans can explain ML models’ decision-making, was limited to feature importance in three of the tree-based models [91,121,132]. We note an absence of commonly used interpretability tools in the literature, such as SHapley Additive exPlanation (SHAP) analysis and permutation feature importance (PFI).

### 3.12. Data and Modeling Availability

Eight studies indicated willingness to share their trained model upon request [95,98,114,115,116,117,119,141]. Three studies have source code available upon request [106,132,133]. Exceptionally, Loer et al., 2023, provided an open-access GitHub repository with full model implementation [95]. Mathematical descriptions for the popPK models were available within the manuscript for 102 studies.

### 3.13. Confidence in Evidence

The assessment of the quality of evidence using GRADE demonstrates a high certainty of evidence for analytical models of tacrolimus dose and concentration prediction, and identification of significant influencing factors on tacrolimus pharmacokinetics characteristics (Supplementary Material S5). Moderate certainty exists for AUC prediction due to concerns regarding patient population bias and lack of generalizability. Low certainty exists for pharmacokinetics parameter prediction studies due to the risk of bias and imprecision.

## 4. Discussion

This systematic review of 115 studies, aggregating different endpoints (AUC, trough, pharmacokinetic parameters, and dose predictions) and time windows, revealed that despite decades of modeling research, widespread clinical adoption of tacrolimus dosing models remains elusive. While popPK models dominate, ML approaches have become increasingly prevalent in the past 5 years (10 of 50 studies published since 2020). No studies employed RL, an approach well-suited for sequential decision-making, despite RL’s success in other therapeutic drug monitoring, such as warfarin (Patel et al.) [145].

### 4.1. Limited External Validation

A significant finding is the lack of external validation through clinical trials and meta-analysis. Despite Shi et al.’s clinical trial demonstrating the superiority of the model-based dosing over clinician dosing in achieving target therapeutic ranges, their small inpatient sample size inhibits generalization [14]. We found that meta-analysis of existing studies could not be completed due to the limited clinical validation, with different endpoints and limited sample sizes within each study.

Only ten studies reported external validation, raising generalizability concerns [18]. Zhao et al. evaluated 16 popPK (dose recommendation for kidney transplant recipients) and showed poor external predictability (F30 under 50%), with improvements when using Bayesian forecasting with 2–3 prior troughs [18]. This likely highlights that models capture training population-specific patterns rather than generalizable ones [18,56].

Among the externally validated development studies, shared characteristics, including larger development cohorts and multi-center populations, potentially contributed to superior transferability. However, standalone evaluation studies, e.g., Methaneethorn et al. [101] in a kidney cohort, or Kirubakaran et al. [19] in heart transplant recipients, demonstrated that external validation success in one population does not guarantee generalizable performance, highlighting that generalizability should be prospectively demonstrated.

### 4.2. Modeling Approaches

popPK modeling techniques are the most prevalent and validated, especially two-compartment models with Bayesian estimation, providing mechanistic insight into tacrolimus kinetics [122]. However, ML methods, especially XGBoost, NN, and hybrid popPK-ML models, have recently gained attention, showing competitive performance. ML approaches can handle high-dimensional, complex, nonlinear relationships without prior assumptions [21,135]. While ML models have been reported to have achieved accurate predictions (Zhang et al.’s TabNet [135] and Huo et al.’s LSTM [146]), these finding have not yet been replicated through external validation and therefore remain far from clinical use.

The head-to-head studies [121,132] compared PK to ML models, showing ML had incremental improvement over Bayesian estimation for dose prediction. Given the marginal improvements in accuracy reported for ML models over PK methods, the trade-off between interpretability and complexity should be considered. ML models demonstrated mechanistically meaningful covariates, which suggest that interpretability loss is marginal compared to the improved accuracy. Nevertheless, ML models incorporating PK models may benefit from both improved performance and interpretability.

### 4.3. Predictive Variables

Strong predictors include CYP3A5 genotypes, hematocrit, POD, and weight. Clinical utility of genetic testing faces significant challenges due to its limited availability and cost [147]. Despite CYP3A5 expressors requiring 1.2–2.2 times higher doses [78,81,82], the clinical impact of genomic testing might not be significant beyond initial dosing [9,148]. Recent studies, like that of Hue et al., achieved a strong predictive performance without genomics, suggesting that prior dose response data accounts for genomic and other variables sufficient to empower day-to-day dose adjustments beyond the first few days of tacrolimus initiation.

Several standard-of-care variables are consistently predictive. Hematocrit affects tacrolimus distribution, as tacrolimus binds to red blood cells [78] and patient weight influences *V_d_* and CL/F [50,54,84,90,102,105]. However, clinicians often adapt individual dosing by accounting for additional factors, such as data collection issues (e.g., sample collection timing mismatch), patient adherence to instructions, social factors, the evolving patient health status, and foreseeable changes to the patient state. These nuanced factors are often only captured in unstructured data (e.g., clinical notes) and are therefore not leveraged by current modeling approaches. No studies incorporated clinical notes or adherence data, despite medication adherence being a known confounding determinant of tacrolimus variability and long-term graft survival [149,150]. For all modeling types, undetected non-adherence is a significant silent predictor, because, for instance, a model will interpret a subtherapeutic trough as requiring an increased dose, whereas the true issue is a missed or late prior dose. Future work could endeavor to quantify adherence in outpatient settings in structured fields using tracking apps or smart medication dispensers, or develop long-acting injectables that can be better controlled. Nevertheless, patient compliance is a well-recognized challenge in medicine that does not have simple solutions.

### 4.4. Outcomes

No studies predicted long-term clinical outcomes such as graft survival. Current models (90 studies) predict short-term targets such as the next-day concentration, whereas the ultimate goal of a transplant is long-term graft survival.

Model calibrations at extreme concentrations were limited [18,39,56,110,135], and the error was not stratified by subgroups [18,39,56,110,135]. While extreme values (demonstrating high-risk zones) represent a small fraction of the cases, these are where clinical consequences may be the most severe. Future work should weigh these extreme events more heavily or employ a priori limits to avoid unintended model predictions and consequences.

Current evaluation methods primarily rely on error-based metrics comparing predicted and observed values. When values are the prescribed dose, error-based metrics reflect the modeling of prescription patterns. For therapeutic drug monitoring in prospective studies, this approach also has limitations, as the target concentration is only a population estimate—not an exact patient-specific target. Alongside error-based metrics, more clinically meaningful evaluations, interventional prospective trials, and clinically relevant therapeutic-based metrics such as F20/F30 or time in the therapeutic range (TTR) [151] should be incorporated for model-based dosing methods. Likewise, in future prospective studies, patient outcomes such as symptoms, survival and quality of life should be considered. These outcome-based metrics should be incorporated alongside error metrics, to improve suboptimal clinical decisions as well as account for long-term transplant outcomes, especially in dose optimization models.

### 4.5. Suggestions for Future Research

Our findings underscore the importance of incorporating clinically relevant covariates in predictive models, including the retrospective dose response, hematocrit, body weight, and POD. Future research should transition to using more holistic evaluation methods to directly measure successful tacrolimus therapy delivery rather than comparing the model to the standard of care. Sequential modeling approaches, such as RL methods [152,153], should be explored to better account for the long-term clinical outcome of a transplant. Most importantly, this review found a lack of external validation and clinical translation. The absence of externally validated models is an important barrier to prospective clinical evaluation [32]. Future research should focus on comprehensive external validation, sequential decision-making modeling, and integrating other real-world clinical factors that influence dosing decisions, including those derived from non-structured clinical reports. To make ML models interpretable, future research should include interpretability tools such as SHAP or PFI when reporting modeling results. Finally, similar to the work of Loer et al., open-source code and implementation should be adopted as best practices in clinical data science and AI.

### 4.6. Limitations and Strengths of This Review

This review was limited to English and French studies. Furthermore, despite our intentions, ultimately, there were insufficient studies for a meta-analysis. However, we found a consistent pattern of increasingly complex modeling methods with no significantly demonstrated clinical benefits, largely due to a lack of external validation or clinical trials. This highlights study challenges rather than methodological limitations, which will require greater collaboration between clinics to overcome. Lastly, we used the JBI Appraisal Tool instead of PROBAST (Prediction model Risk of Bias ASsessment Tool) as we explored a variety of study designs for the prediction models.

## 5. Conclusions

Tacrolimus concentration predictions and dosing recommendations are commonly explored using population-based pharmacokinetic models, with recent ML approaches showing promise for incremental improvement. The field is currently limited by minimal demonstration of generalizability through external validation. Future models may benefit from incorporating underutilized data sources such as patient adherence, exploring sequential decision-making models like reinforcement learning, and modeling beyond the critical period of the first three months post-transplant to account for long-term transplant outcomes. Lastly, this field could benefit from reproducibility through the open sharing of data and models.

Ultimately, for clinical translation, modeling approaches should demonstrate a strong performance in multicenter clinical trials across different populations. To achieve this, there needs to be standardized external validation focused on the clinically relevant performance. Lastly, integration with electronic medical record systems is essential to enable efficient clinical implementation.

## Figures and Tables

**Table 1:** Single-compartment pharmacokinetic parameters’ definitions.

Pharmacokinetic Parameter	Definition
Volume of Distribution (*V_d_*)	The theoretical volume into which the drug is distributed (e.g., plasma) [mL].
Elimination Rate (*k*)	The relative rate at which the drug is removed from *V_d_* [1/min]. The absolute elimination rate typically decreases with the concentration of the drug.
Half-life (t_1/2_)	The time for the drug to decrease by half [min], and *t*_1/2_ = ln2k.
Clearance Rate (*CL*)	The rate at which a volume is cleared of the drug *CL* = *k*·*V_d_* = *k*/*C* [mL/min]. This rate is constant regardless of drug concentration.
Concentration (*C*)	The amount of drug in the *V_d_* [g/mL]. For most drugs, if we know the concentration in the blood at a certain time (*C*_0_), we can model the concentration at any future time (*t*) as $C\left(t\right)=C_{0}\cdot e^{-kt}.$
Area Under the Curve (AUC_x_)	The integral of the blood concentration over a finite time interval x. Because the concentration of tacrolimus changes with a t_1/2_ in the order of hours and dosing is once or twice daily, the AUC is a better clinical descriptor than a single concentration measurement. However, AUC measurements are not routinely viable, especially in the outpatient setting, as they require multiple blood draws between doses.

**Table 2:** Characteristics of the included studies. Gradient Boosted Decision Tree (GBDT), Random Forest (RF), support vector regression (SVR), K-nearest neighbor (KNN), Least Absolute Shrinkage and Selection Operator (LASSO) regression, ridge regression (RR), linear regression (LR), TabNet (Tabular Network), multiple linear regression (MLR), Limited Sampling Strategies (LSSs), Bayesian estimation (BE), multivariate linear regression (MLR), artificial neural network (ANN), regression tree (RT), multivariate adaptive regression splines (MARSs), boosted regression tree (BRT), Bayesian additive regression trees (BARTs), physiologically based pharmacokinetic (PBPK), multilayer perceptron regression (MLP), one-compartment (1CMT), two-compartment (2CMT), mean error (ME), mean absolute error (MAE), mean relative error (MRE), root mean squared error (RMSE), prediction error (PE%), absolute prediction error (APE%), individual PE% (IPE%), median IPE% (MIPE%), median absolute IPE% (MAIPE), F20 of IPE% (IF20%), F30 of IPE% (IF30%), standard error (SE%), %PRED20 (percentage of measured blood levels predicted within a 20% interval), mean relative deviations (MRDs), geometric mean fold errors (GMFEs), bias (median percentage predictive error), imprecision (median absolute percentage predictive error), root-mean-squared error of cross-validation (RMSECV), goodness-of-fit plots (GOF), visual predictive checks (VPCs), Therapeutic Drug Monitoring (TDM).

Primary Author, Publication Year	Setting	Study Method	Transplant Organ	Time Since Transplant	Sample Size [Dev/Val]	Age [Dev/Val]	%Male	Modeling Technique	Predicted Target	Outcome	Performance Metrics
Abderahmene, 2024 [39]	Tunisia	Retrospective study	Kidney	First 3 months	337, 196	56.25, 39.43 [Mean]	6.53, 72.44	popPK (2CMT with first-order absorption)	Initial dose	Accurate prediction of the target, found out CYP3A and age have a small effect on tacrolimus clearance in the validation cohort but significant differences between the two cohorts	GOF, VPC
Al-fokahi, 2021 [40]	USA	Multi-center observational	Kidney	Varying	608, 1361	52, 52	62.62	popPK	C/D prediction, significant predictors	Genotype significantly affects TAC pk; CYP3A5, CYP3A4, corticosteroids, calcium channel blocker and antiviral drug use, age, and diabetes significantly contributed to the CL/F	ME, MPE, RMSE
Allard, 2019 [41]	France	Prospective multi-center randomized study	Liver	Day 7 and day 90	12, 12	57, 59 [Median]	83	popPK (2CMT with linear elimination and a delayed first-order absorption with two transit compartments)	PK parameters (early vs. late stages)	Switching from Prograf to Advagraf in early stages could modify calcineurin activity (unrelated) to Tac Pk/PD; no statistical difference in trough in D90 and D104	Diagnostic plots, GOF
Andreu, 2015 [42]	The Netherlands	Retrospective study	Kidney	<1 year	16, 91	56, - [Median]	62.5	popPK (2CMT), BE (LSS)	C/D prediction, significant predictors	Accurate prediction of the target, popPK provides accurate prior information for MAP-Bayesian that predicts accurate AUC	ME, RMSE, bootstrap (200 runs), MPE%
Andreu, 2017 [43]	Spain	Retrospective study	Kidney	7–365 days	304, 59	52, 53 [Median]	60.88	popPK (2CMT with first-order absorption and a lag time), BE	Significant predictors, initial dose	Accurate prediction of the target (individual CL values); one-/two-/three-compartment models were tested; CYP3A5 and CYP3A, age, and hematocrit were significant predictors of Tac	MPE, RMSE, VPC, bootstrap (200 runs)
Andrews, 2019 [44]	The Netherlands	Retrospective study	Kidney	<3 months	337, 304	56.95, 52 [Median]	60.5, 65.8	popPK (2CMT with first-order absorption)	Initial dose	Higher body surface area, lower creatinine, younger age, higher albumin and lower hematocrit also resulted in higher tacrolimus CL/F; starting dose should be increase by 160% in CYP3A5 carriers and reduced by 80% in non-carriers	VPC
Antignac, 2005 [45]	France	Retrospective study	Liver	11–66 days	37, -	52, - [Median]	70.27	popPK (1CMT with linear absorption and elimination), BE	Significant predictors	Very low dose should be administered post Tx, dose can be increased once CL/F increases; Bayesian estimation performs best >15 days post-transplant and shows large interindividual variations in CL before that	GOF, bootstrap (1074 runs), RMSE
Antignac, 2011 [46]	France	Retrospective study	Kidney	Varying	33, -	51, - [Mean]	84.84	popPK (1CMT with linear absorption and elimination), BE	Trough	Bayesian method can predict concentration with a few of samples	ME, MAE, RMSE
Åsberg, 2013 [12]	Norway	Retrospective study	Kidney	N/A	69, 30	41, 57 [Median]	71	popPK (3CMT with first-order absorption and lag time)	Significant predictors, nest dose	CYP3A5 genotype is influential and improves dose predictions and increases CL/F; CYP3A5 improves the predictions but the model does equally well when having 3–4 trough concentration in CYP3A5 absence	PE, mean weighted PE, RMSE, R2, slope of the individual predicted versus observed plots
Barraclough, 2011 [27]	Australia	Retrospective study	Kidney	Varying	20, Jackknife	49, - [Mean]	60	BE (LSS)	AUC0-12	Limited sampling method is better predictor than Bayesian method for C0 based on AUC0-12, C0 is poorly correlated with AUC0-12	MPE, MPPE, RMSE, MAPE
Barraclough, 2012 [47]	Australia	Retrospective study	Kidney	10 patients: week 1, 10 patients: >3 months	20, Jackknife	49, - [Median]	60	MLR (LSS)	AUC0-12	Accurate prediction of the target using C0.5, C2, and C4 for early and late stages, these sampling times can be used for tacrolimus, mycophenolic acid and unbound prednisolone AUC	Jackknife, MPE, MPPE, RMSE, MAPE
Barraclough, 2022 [48]	Australia	Multi-center, prospective, observational study	Kidney	>1 month (<5 year)	43, -	53.6, - [Median]	46.51	popPK (2CMT with first-order absorption and a lag time), BE	Significant predictors	There is no difference in typical tacrolimus pharmacokinetics between Aboriginal and Caucasian recipients; greater between-patient variabilities in CL/F in Aboriginals	OFV, GOF
BenFredj, 2016 [49]	Tunisia	Retrospective study	Kidney	≤3 months, 3–12 months, >12 months	50, 26	30.21, 33.25 [Mean]	70	PK (non-parametric adaptive grid approach)	C0/D prediction	Age, sex and weight do not significantly affect CL/F and *V_d_*; showed there is a significant increase in C0/D within the first year	GOF, MPE, RMSE
Ben-Fredj, 2020 [50]	Tunisia	Cross-sectional	Kidney	≥1 day	77, 25	33.5, - [Mean]	68.6	MLR	C/D prediction, significant predictors	POD, CYP3A4 and 5 are associated with C/D; sCYP3A4 is associated with lower Tac exposure	R2, Bland–Altman
Ben-Fredj, 2023 [51]	Tunisia	Prospective single-center study	Kidney	N/A	97, 71	36.4, - [Mean]	N/A	BE	C/D prediction	Model cohort was nearly twice as likely to have C0 within the range	R2, MAPE, RMSE, Bland–Altman
Benkali, 2009 [52]	France	Retrospective study	Kidney	Week 1 to month 6	32, Bootstrap (500 runs)	54, - [Mean]	59.3	popPK (2CMT with Erlang absorption and 3 delay compartments), BE	AUC0-12, significant predictors	Accurate prediction of the target using C0, C1 and C3; hematocrit and PXR genotype are covariates on CL/F	Bias, RMSE, comparing observe and predicted
Benkali, 2010 [53]	France	Retrospective study	Kidney	≥12 months	29,12	52, - [Median]	46.34	popPK 2CMT with Erlang absorption and 3 transit compartments, BE (LSS)	pk parameters, AUC0-24	Accurate prediction of the target using C0, C1, and C3; CYP3A5 is a significant covariate on CL/F	Bootstrap (1000 runs), VPC
Birdwell, 2012 [54]	USA	Retrospective study	Kidney	Varying	446, -	46, -	61.4	MLR, longitudinal data analyses	C/D prediction, significant predictors	Increase in albumin and weight found to be associated with decrease in C/D ratio while age increased it; model predictive accuracy increases in presence of genetic variants	N/A
Brooks, 2021 [30]	Australia	Retrospective study	Kidney	>1 month	20, 0	52.5, - [Median]	45	BE (LSS)	AUC0-12	All 3 Bayesian forecasting programs/services evaluated had reasonable performance when using C0, C1, and C3	MPE, MPPE, RMSE, MAPE
Cai, 2020 [55]	China	Retrospective study	Kidney	≥3 months	182, -	37.9, - [Mean]	74.7	RF	C/D prediction, next dose	Accurate prediction of tacrolimus metabolism and dose requirements; CYP3A4 is more significant than CYP3A5 for TAC disposition	MDPE, MAPE, PE%
Cai, 2020 [56]	China	Retrospective study	Liver	4–50 days	84, -	51, - [Mean]	82.1	popPK (nonlinear Michaelis–Menten), BE	Significant predictors, next dose	popPK MM outperformed linear one- and two-compartment models with first-order elimination meaning TAC pk are nonlinear; Bayesian form acting significantly improved predictions	RMSE, R2, RMSECV, the square correlation coefficients of cross-validation
Cai, 2022 [57]	China	Retrospective study	Liver	2–72 days	176, -	50.68, - [Mean]	85	popPK (1CMT with first-order absorption and elimination), nonlinear Michaelis–Menten (MM)	pk parameters	Nonlinear MM was superior to the PK model and better described pk behavior; tacrolimus concentration to dose and metabolism may contribute to nonlinear behavior	GOF, MDPE%, MAE%
Catic-Dordevic, 2018 [28]	Serbia	Retrospective study	Kidney	N/A	20, 16	38.2, 40.6 [Mean]	50	Monte Carlo simulation (with respect to gender)	AUC0-12	Gender-specific sampling based on MC simulations are C1 and C8 post-dose for females and C8 for males	prediction error (MAPE%)
Chen, 1999 [58]	USA	Retrospective study	Liver	<6 months	10, 22	46.13, 48.8 [Mean]	68.75	NN combined with Genetic Algorithm	Trough	NN can predict whole-blood concentration but requires a large retrospective dataset to train on	R, R2, Bootstrap (10,000 runs)
Chen, 2005 [59]	China	Retrospective study	Kidney	N/A	16, -	38.3, - [Mean]	75	Multiple stepwise regression analysis	AUC0-12	Accurate prediction of the target using C5, C1.5 (and C3); C5 might be the best single point to guide TAC dose	Predicted vs. observed mean (+/− SD)
Chen, 2017 [60]	China	Retrospective study	Liver	>4 days	125, -	47.6, - [Mean]	82.4	popPK (2CMT with lag time), BE (LSS)	Significant predictors, AUC0-12	CYP3A, creatinine clearance and POD are found to be significant covariates of CL/F; AUC can include C0 and C2 (and C4); found that the best structural model for C0 is a 1-compartment model by the first-absorption process without lag time, and for full PK data, a two-compartment model by a single first-absorption process with lag time	R, R2
Chen, 2021 [61]	China	Retrospective study	Kidney	0–90 days	142, Bootstrap (1000 runs)	40.9, - [Mean]	67.6	popPK (1CMT with the first-order absorption)	Drug–drug interaction	Wuzhi capsule in low dosage exerts the optimum effect of tacrolimus	GOF, bootstrap (1000 runs), VPCs, and normalized prediction distribution errors (NPDEs)
Choshi, 2024 [62]	Japan	Retrospective study	Lung	N/A	119, 6	N/A	N/A	Multivariate LSTM	C0	Dose, route and C0 are the most important variables in predicting future trough	Accuracy, actual vs. predicted plot
Damon, 2017 [63]	France	Retrospective study	Kidney	10–90 days	280, 189	N/A	N/A	Partial Least Squares regression multivariate predictive	Significant predictors (Genomics)	Up to 70% dose variability can be predicted by metabolism enzymes and transporters	R2, SE, Bootstrap (1000 runs)
Dansirikul, 2004 [64]	Australia	Retrospective study	Liver	11–1886 days	31, Jack-knife	47, - [Mean]	74.19	popPK (2CMT with first-order absorption and first-order elimination), noncompartmental model (LSS)	AUC0-6, AUC0-12	Best sampling times using regression equations were at C2, C4, and C5 rather than through; C5 is the most informative sampling time for AUC0-12	R2, ME, RMSE, jackknife
Decrocq-Rudler, 2021 [65]	France	Retrospective study	Liver	1–77 days	55, 24	55.2, 58.6 [Mean]	63	popPK (1 and 2CMT models with first-order elimination), BE	Next dose	Accurate prediction of the target using Bayesian forecasting	MDPE, MDAPE, PE, F20, F30
Du, 2022 [66]	China	Retrospective study	Liver	2–538 days	116, 29	49.43, 51.31	72.41	popPK (1CMT with first-order absorption and elimination)	Significant predictors, next dose	Accurate prediction of the target, Wuzhi capsule, POD, eGFR, hemoglobin and albumin are associated with CL/F, and ALT and UREA are among the variables affecting V/F	PE, MDPE, MAPE
Du, 2024 [67]	China	Retrospective study	Lung	N/A	210, -	60, - [Median]	79.5	Linear regression analysis	C/D prediction, significant predictors	Four SNPs are identified to be associated with Tac metabolism, accurate prediction of the initial dose using the model	MAPE, F20, F30
Du, 2024 [68]	China	Retrospective study	Liver	85.5–20 days	31, (232, 57 concentration profiles)	50.90, 50.84 [Mean]	70.96	ANN	Trough	Accurate prediction of the target; performs better than popPK models; daily dose, recipient age, recipient and donor CYP3A5 are significant influencers of TAC concentration	PE ± 30%, Bootstrap (1000 runs)
Elens, 2011 [69]	Belgium	Cross-sectional	Kidney	≥2 years	99, -	50.5, - [Mean]	62	Linear and logistic regression	Significant predictors	CYP3A4 is more influential on tacrolimus metabolism than CYP3A5	PE, mean prediction error (MPE) and mean absolute prediction error (MAE), R2
El-Nahhas, 2022 [70]	UK	Retrospective study	Kidney	>2 years	15, 9	52.75, - [Mean]	75	Multiple linear regression (LSS)	AUC0-24	Accurate prediction of the target using C2 and C10 (and C0); LSS using C0 alone is suboptimal	AIC, R2, ROC curve
Faelens, 2022 [71]	Belgium	Retrospective study	Kidney	0–14 days	315, -	53, - [Median]	63.4	popPK (1CMT with oral absorption)	Dose prediction (early stages)	Improved dosing when using a model, no real improvement from a 2-CMT model or from the incorporation of available covariates (hematocrit)	R2, Partial R2
Francke, 2022 [72]	The Netherlands	Prospective single-arm clinical trial study	Kidney	Varying	59, -	59, - [Median]	62.7	BE	Initial dose	Observed vs. model-based did not differ significantly, with model-based dosing being slightly better, while model-based simulation showed lower interpatient variability and higher target achievements; a combination of an algorithm starting dose + model-based follow-up has potential to reduce adverse effects	PE, MPE%, MAE%, RMSE
Francke, 2022 [73]	Germany	Retrospective study	Kidney	N/A	46, -	65, - [Median]	52	popPK (2CMT with 1st-order absorption and lag time)	PK prediction, dose	Body composition is associated with tacrolimus pk and can improve its dose requirements; phase angle is positively correlated with TAC pk	PE%, VPC, GOF
Fu, 2022 [74]	China	Retrospective study	Kidney	1.5–20.5 days	2040, 511	39.72, 38.73 [Mean]	64	popPK, AdaBoost regressor, bagging regressor, DT, KNN, LASSO, MLP, SVR, RF	Next dose	Extra Trees Regressor accurately predicts the target	GOF, VPCs, bootstrap (1000 runs)
Gaies, 2013 [75]	Tunisia	Retrospective study	Kidney	N/A	20, Bootstrap (1000) and Jackknife	31, - [Median]	95	popPK (2CMT with Erlang distribution to describe the absorption phase and three delayed compartments), Bayesian estimation	AUC0-12	Accurate prediction of the target using C0, C1 and C3	PE, counts in therapeutic range
Gérard, 2014 [76]	France	Open-label, non-comparative, prospective, observational study	Liver	1–25 days	66, -	52.9, - [Mean]	N/A	PBPK (13CMT)	Initial dose	Order of covariates that impact TAC C0: plasma unbound tacrolimus, typical intrinsic clearance, bioavailability, body weight, hematocrit, CYP3A5 polymorphism, proportion of fat, and CYP3A4 inhibitory drug–drug interactions; proposed C0 as a function of CYP3A5 donor genotype and patient’s hematocrit and body weight	Percentage within 20% of actual dose, R2
Grover, 2011 [77]	USA	Retrospective study	Kidney	7–53 months	24, -	52, - [Mean]	63	popPK (2CMT with first-order absorption and lag time), BE (LSS)	pk parameters, significant predictors	Native Americans show lower clearance compared to whites; therefore, they may require lower doses to avoid toxicity	OFV, GOF
Han, 2014 [78]	Republic of Korea	Retrospective study	Kidney	≥2 weeks	122, -	41.9, - [Mean]	54.91	popPK (1CMT with first absorption and elimination and lag time), BE	Trough, significant predictors	CYP3A5 genotype and POD are significant predictors in early stages; CYP3A5 influences CL/F and POD decreases CL/F	GOF, Bootstrap (2000 runs), VPC (1000 runs)
Han, 2019 [79]	China	Retrospective study	Heart	4–41 days	107, 24	51, 40 [Median]	81	popPK (1CMT with first-order absorption and elimination), BE	C/D prediction, significant predictors	CYP3A5 genotype is influential and requires higher dose than nonexpressers; CL/F was significantly reduced in CYP3A5 nonexpressers, with Wuzhi capsules and with antifungal meds	GOF; Bootstrap (1000 runs), VPC
Itohara, 2022 [80]	Japan	Retrospective study	Kidney, Liver	≥3 weeks	18, - renal, 13, - liver	51.2, - Kidney, 58, - Liver	55.5, 53.8	PBPK (15CMT)	AUC0-12	PK model derived in kidney transplant patients was applicable to liver transplant patients	PE%
Ji, 2018 [81]	Republic of Korea	Retrospective study	Liver	14 days	58, -	49.2, - [Mean]	79	popPK (1CMT with first-order absorption and elimination)	pk parameters, next dose	POD and CYP3A5 affect CL/F in living donor recipient	VPC
Jing, 2021 [82]	China	Retrospective multi-center study	Kidney	>1 month	165, -	40.5, - [Mean]	66	popPK (1CMT with first-order absorption and elimination)	Drug–drug interaction	Clearance rate of Tac decreases when combined with Wuzhi capsule; hematocrit, POD and CYP3A5 had significant influence on CL/F	GOF, Bootstrap (1000 runs), VP
Kim, 2012 [83]	Republic of Korea	Retrospective study	Kidney	0–12 months	132, -	38.6, - [Mean]	59.84	Linear mixed-effect modeling	Significant predictors	Age, body weight, hematocrit, serum creatinine levels, and CYP3A5 genotypes were found to be significant factors affecting trough; cadaveric transplantation is associated with increased risk of rejection	95% CI
Kim, 2012 [84]	Republic of Korea	Retrospective study	Kidney	1–5 years	129, -	38, - [Median]	56.6	Linear mixed-effect modeling, multivariate Cox proportional hazards	Significant predictors	CYP3A5 is a variable marker for dose requirements, influencing factors vary depending on different post-transplant periods	95% CI
Kim, 2019 [85]	Republic of Korea	Retrospective study	Kidney	0.6–10.4 years	32, Bootstrap (1000 runs)	52, - [Median]	63	popPK (two-compartment with first-order absorption with lag time, and first-order elimination)	pk parameters, significant predictors	CYP3A5 and MMF significantly affect TAC CL/F, effect of MMF on TAC exposure is more pronounced in CYP3A5 non-expressors	Bias, imprecision
Kirubakaran, 2022 [19]	Australia	Retrospective study	Heart	≤391 days	85, -	55, - [Median]	67	popPK (1 and 2CMT with first-order absorption—validating other models on this data)	Next dose	Failed to predict the target for the heart transplant recipients using popPK models developed from various solid organ transplant recipients	GOF, bias, imprecision
Kirubakaran, 2023 [86]	Australia	Retrospective study	Heart	N/A	47, 40	53, 56	67	popPK (2CMT with first-order absorption), BE	Significant predictors, PK parameters	Accurate prediction of the target accounting for azole antifungal medication; concomitant azole antifungal therapy reduced tacrolimus CL/F by 80%; recent tacrolimus concentration is sufficient for predicting PK parameters	Bias, imprecision, Bootstrap (1000 runs)
Kirubakaran, 2024 [26]	Australia	Retrospective study	Lung	90 days	43, -	N/A	N/A	popPK (various models), Bayesian estimation	C/D prediction, significant predictors	Models developed for non-lung recipients perform poorly on lung cohort when concomitant antifungal therapy was present, but showed potential applicability in absence of concomitant antifungal therapy	R2, Odds Ratio, 95% CI
Kuypers, 2004 [87]	USA	Open-Label, retrospective study	Kidney	N/A	100, -	51.4, - [Mean]	59.12	Multivariate logistic regression	Significant predictors (in terms of rejection rates)	Increasing serum albumin and hematocrit concentrations were associated with a prolonged concentration and contributed to lower risk of rejection; suggest shorter transit time of tacrolimus in certain tissue compartments, rather than failure to obtain a maximum absolute tacrolimus blood concentration, might lead to inadequate immunosuppression early after transplantation	R2, MAPE%, MPE%
Langers, 2008 [88]	The Netherlands	Retrospective study	Liver	≥6 months	23, -	44.47, - [Mean]	47	popPK (2CMT with first-order absorption without a lag time) (LSS)	AUC0-12	Accurate prediction of the target using C4 and C6; C0 is not an accurate way of assessing systemic exposure for either formulation	ME, MAE, RMSE
Li, 2007 [89]	China	Retrospective study	Liver	Varying	72, 32	49, 51 [Median]	86.53	popPK (1CMT with first-order absorption and elimination)	Significant predictors	Total bilirubin and CYP3A in both donor and recipient are found to be significant variables on CL/F	SE
Li, 2011 [90]	China	Retrospective study	Kidney	N/A	142, -	42.6, - [Mean]	69.7	Multiple stepwise linear regression analysis	Next dose, significant predictors	Accurate prediction of the target, confirm CYP3A5, body weight, hematocrit, hemoglobin and total bilirubin significantly impact dose maintenance	PE%, MPE, RMSE, F30%
Li, 2023 [91]	China	Retrospective study	Liver	N/A	145, 36	51, 51 [Median]	85.63	popPK (1CMT with first-order absorption and elimination), BE, XGBoost	C0	Combining popPK and XGBoost might improve trough prediction, XGBoost shows minimum MPE, popPK + ML can improve predictions	GOF, MPE, MAE, Bootstrap (2000 runs), normalized prediction distribution errors (NPDEs)
Ling, 2020 [92]	China	Retrospective study	Kidney	0–30 days	234, 18	39, 40 [Median]	69	popPK (1CMT with first-order absorption and elimination)	Significant predictors	CYP3A5 genotype, POD and hematocrit are significant predictors and affect CL/F in early stages	R2, PE, APE, MPE MAPE, Bland–Altman plot
Liu, 2020 [93]	China	Multi-center retrospective study	Liver	<6 months	373, -	51, - [Median]	78	MLR	Initial dose, significant predictors	Both donor and recipient CYP3A5 genotypes influence C/D ratio in early stages (3-month post) and donor is of primary importance	MPE), MAPE
Lloberas, 2023 [94]	Spain	Prospective controlled, 2-arm, randomized, open-label, single-center trial	Kidney	Days 5, 10, 15, 30, 60, 90	48, 42	63.5, 63.5 [Median]	73.3	popPK	Next dose	Pk-based dosing resulted in more significant TTR compared with the control group	Residual plots, Shapiro–Wilk test, IQR, *p*-value, ME, SE
Loer, 2023 [95]	Germany	Retrospective study	Varying	Varying	700, 300	35, 35 [Mean]	N/A	popPK	Food/drug–drug interaction	CYP3A4 is more influential on tacrolimus metabolism than CYP3A5	GOF, MRDs, GMFEs
Macchi-Andanson, 2001 [96]	China	Retrospective study	Liver	First 2 weeks	40, -	48, - [Mean]	72.5	popPK (1CMT with first-order absorption, first-order elimination), BE	Next dose	As the model poorly predicted the target, it is suggested that trough levels are not proper predictors of individual dosing	GOF plots, R, ME, RMSE, PRED20%
Marquet, 2018 [97]	France	Multi-center, prospective, randomized, open-label, parallel group study	Kidney	Day 8, Day 90	44, -	51.8, - [Mean]	75	popPK (1CMT open with two gamma absorption laws), Bayesian estimation (LSS), MLR	AUC0-24	Similar exposure in both formulations, CYP3A5 explains ~31% of the variability in AUC, no influence of gender; AUC0-24 is more correlated with C24 than C0, AUC-to-trough level ratio was similar in both formulations	Mean relative bias, RMSE, VPC
Marquet, 2021 [98]	France	Multi-center retrospective study	Kidney	Day 7, month 1, and month 3	29, 7	59, 59 [Median]	75.86	popPK (1CMT with double gamma absorption, linear elimination), BE (LSS)	AUC0-12	Accurate prediction of the target using C0, C1 and C3, no need for CYP3A5 genotype for modeling AUC	RMSE, Bland–Altman plots, number of differences out of the ± 20% acceptable range, R2
Mathew, 2008 [99]	India	Retrospective study	Kidney	3–6 months	29, Jack-knife	32, - [Mean]	82.75	LSS	AUC0-12	Accurate prediction of the target using C0 and C1.5, which performed better than C0 and C4, marginal R2 improvement when more concentration samples added	PE%, APE%
Matsuda, 2022 [100]	Japan	Retrospective study	Lung	N/A	20, -	44.5, - [Median]	55	Linear mixed-effect modeling	Drug–drug interaction	C/D ratio increased by 2.25-fold when co-administered with itraconazole; CYP3A5 could contribute to interindividual variability	GoF, AIC
Methaneethorn, 2022 [101]	Thailand	Retrospective study	Kidney	≥51 days	74, -	45.79, - [Mean]	60.81	popPK (2CMT first-order, Erlang distribution, or transit compartment absorption), BE	Next dose	Accurate prediction of the target using Bayesian post hoc estimation	MAPE, RMSE, MSE, 95% CI
Moes, 2016 [102]	The Netherlands	Retrospective study	Liver	N/A	66, Bootstrap (1000 runs)	54, - [Mean]	62.5	LSS, popPK (2CMT with first-order elimination and h delayed absorption)	Significant predictors, AUC0-24	Accurate prediction of the target using 3 blood samples at C0, C2 and C3; age, weight, sex, hematocrit, hemoglobin, albumin, creatinine, BSA, BMI, LBW, co-medication, primary diagnosis, and ethnicity are not significant on CL/F, V/F or K	95% CI, MPE, MAPE, and RSME, R2, Bootstrap (1000 runs)
Musuamba, 2009 [103]	Belgium	Retrospective study	Kidney	Varying	19, -	42, - [Median]	84	popPK (2CMT with first-order absorption and elimination)	Significant predictors	Time of drug administration significantly impacts absorption rate constant, absorption rate varying in day vs. night administration, Circadian variation in tacrolimus absorption will not modify patient outcomes	BIC, Bland–Altman analyses, RMSE, MRPE
Musuamba, 2013 [104]	Belgium	Retrospective study	Kidney	0–1 month	65, -	N/A	N/A	MLR, BE	Significant predictors, AUC0-12	Age, co-medications and time post-transplantation are reported to be significant predictors, Bayesian estimator performed better than MLR, C1.5 and C3.5 showed best predictive performance	R2, RMSE, PE, Bland–Altman
Nanga, 2019 [105]	France	Retrospective study, systematic review	Kidney, liver, lung, and HCT	Varying	281, -	2.3, - [Median]	37	popPK (2CMT with first-order absorption, absorption lag time and first-time varying elimination); external validation	Next dose (early stages)	Model validated across different age groups and organ transplants; post-operative time influences drug CL, whereas body weight influences *V_d_* and CL	Diagnosis scatter plots, VPC, bootstrap
Nguyen, 2023 [106]	USA	Retrospective, cross-sectional, single center, open-label study	Kidney	≥6 months	67, 15	49.05, 57.2 [Mean]	58.5	popPK (2CMT model with first-order absorption and elimination with an absorption lag-time), BE	AUC0-12	Accurate prediction of the target; to improve predictions, there needs to be more longitudinal and biological data for modeling	Bland–Altman plots, rRMSE, rBias, R2
Niioka, 2015 [107]	Japan	Retrospective study	Kidney	0–30 days	50, -	50.5, - [Mean]	68	MLR, PK (non-compartmental)	Significant predictors	CYP3A5 genotype is influential after day 14 post-transplant	R2, Bias, SE, bootstrap
Op den Buijsch, 2007 [25]	The Netherlands	Retrospective study	Kidney	>12 months	37, -	51.03, - [Mean]	65	Regression equation (limited sampling)	AUC0-12	Accurate prediction of the target, C0 and C12 have a lower predictive value for AUC0-12, LSS based on regression analysis is superior to LSS based on Bayesian fitting	PE%, APE%, R2
Oteo, 2013 [108]	Spain	Retrospective study	Liver	0–14 days	75, -	N/A	N/A	popPK (1CMT with first-order absorption), BE	Next dose	Accurate prediction of the target when combined with individualized biochemical assay	MPE, RMSE
Pankewycz, 2020 [109]	USA	Retrospective observational study	Kidney	≤12 months	113, -	49, - [Median]	58	Scoring formula (LSS) [TAC TDM × (MPA AUC + MPAG AUC/10)]	Stable, over-/underexposure score	Scoring method accurately categorizes patients 6–12 months post-Tx into 3 categories	ROC analysis
Pei, 2023 [110]	China	Retrospective study	Heart	<1 month	115, -	52, - [Median]	N/A	popPK (15CMT with first-order absorption)	pk parameters	Accurate prediction of the target; hematocrit should be considered as a significant influencer on C0 and AUC	
Ragette, 2005 [111]	Germany	Retrospective study	Lung	3–18 months	15, -	42.0, - [Mean]	53	Linear analysis	AUC0-12	Accurate prediction of the target using recommended C0/C4, C2/C4, and C0/C2/C4; using at least 2 or 3 concentrations between 0 and 4 h post-drug is required; true TAC exposure proved highly variable and a poor predictor of C0	R2, 90% CI, fold error (predicted value/observed value)
Resendiz-Galvan, 2019 [112]	Mexico	Retrospective study (observational and mainly ambispective)	Kidney	4–2730 days	52, 13	36, 33 [Mean]	61	popPK (1CMT first-order conditional estimation method with interaction)	Next dose, significant predictors	Accurate prediction of the target; hematocrit and CYP3A5 significantly affected CL/F	APE, R2
Riff, 2019 [113]	France	Retrospective study	Liver	Day 7 and week 6	80, -	41.5, -	-	popPK (1CMT with first-order elimination and 2 γ-distributions), BE	AUC and dose prediction	Accurate prediction of the target using C0, C1 and C6 for Advagraf, C0, C2 and C6 for Prograf on Day 7 and C0, C1 and C3 in Week 6	Observed versus individual predicted concentration plots, weighted residual error versus individual predicted concentration plots, visual predictive checks (VPCs) (1000), 90% prediction intervals
Rong, 2019 [114]	Canada	Retrospective study	Kidney	<100 months	49, -	50, - [Mean]	44.89	popPK (1CMT with first-order absorption with a lag time, linear elimination, and constant error)	Significant predictors	Accurate prediction of the target regardless of the post-transplant period; eGFR had significant effect on Cl	GoF, VPC, Bootstrap (500 runs), 95% CI
Saint-Marcoux, 2005 [115]	France	Retrospective study	Lung	N/A	22, -	40, -	50	popPK (1CMT with first-order elimination and double gamma absorption), BE	AUC0-12	Accurate prediction of the target using 3 blood samples at C0, C1 and C3 for non-cystic fibrosis (CF) and C0, C1.5, and C4 for CF patients	Mean bias, RMSE
Saint-Marcoux, 2010 [116]	France	Retrospective study	Kidney	Day 14 and Day 42	12, -	N/A	N/A	popPK (1CMT with absorption described as following a double gamma distribution), BE	AUC0-24	Accurate prediction of the target using C0 and C0/dose	Observed vs. estimated concentrations, Bayesian AUC0–24h estimates of LSS vs. linear trapezoidal rule applied to the full profiles (reference values), bias, RMSE
Saint-Marcoux, 2011 [29]	France	Retrospective study	Kidney	>12 months	45, -	N/A	N/A	popPK (1CMT model with first-order elimination combined with a gamma model of absorption with 2 parallel absorption routes); BE	Dose prediction, AUC0-24	Accurate prediction of the target; analytical method impacts the performance of Bayesian estimation	Mean bias +/− SD between observed and modeled concentrations, RMSE, squared correlation coefficients between observed and modeled concentrations, mean bias 6 SD between trapezoidal and Bayesian AUC0–24 h (extreme values)
Saint-Marcoux, 2013 [117]	France	Multi-center retrospective study	Kidney	Varying	1000	47.5, - [Mean]	N/A	Regression analysis	AUC vs. C0	C0 and AUC strongly linked in 1st 3 months after transplant; after 3 months, the relationship remained significant but was weaker	Predicted vs. observed, R2
Scholten, 2005 [118]	The Netherlands	Retrospective study	Kidney	2–52 weeks	17, 26	45.4, 46.9 [Mean]	65	popPK (2CMT with a lag time and first-order absorption)	AUC0-12	Accurate prediction of the target using C2 and C4	R2, MPE%, MAPE%
Shi, 2023 [14]	China	Retrospective study + multi-center, randomized, single-blind clinical trial study	Liver	>28 days	150, 79 (40 pilot trial)	48, 50, [Median]	82	popPK (2CMT with first-order absorption)	Next dose	Accurate prediction of the target; model improved initial dose accuracy and reduced the number of adjustments, model-based doses were significantly individualized	Scatter plot, ROC curve
Smith, 2023 [119]	USA	Prospective study	Kidney	>12 months	15, -	N/A	N/A	BE vs. non-compartmental popPK	AUC0-12	MAP-Bayesian estimates the target using 9 sparse samples, comparable to NCA, which also uses 9 samples	RMSE, relative bias
Stifft, 2020 [120]	France	Retrospective study	Kidney	6 weeks and >6 months	27, 24	49, 55, unknown [Mean]	56.86	popPK (2CMT first-order absorption and elimination and with a lag time), LSS (MLR)	AUC0-24	Accurate prediction of the target using C8 via LSS (for early post-transplant)	R, R2
Storas, 2022 [121]	Norway	Retrospective study	Kidney	Varying	68, 7	55, 60 [Mean]	77	XGBoost	AUC0-24	Accurate prediction of the target using C2, C2.5, C3, C4, and C5	MPE%, MAPE%, RMSE%
Storset, 2014 [122]	Australia and Norway	Retrospective study	Kidney	≤21 days	242, 72	48, 53 [Mean]	68	popPK (2CMT with first-order absorption and a lag time)	Significant predictors	Accurate prediction of the target using a theory-based popPK model rather than the empirical models	RMSE, PE
Tang, 2017 [21]	China	Retrospective study	Kidney	N/A	838, 207	36.19, 35.82 [Mean]	71.3	MLR, ANN, RT, MARS, BRT, SVR, RF, LASSO, BART	Next dose	Accurate prediction of the target using all the models, RT performed the best	MPE%, Bootstrap (10,000)
Tornatore, 2022 [13]	USA	Cross-sectional, open-label single center	Kidney	≥6 months	65, -	48.88, - [Mean]	55.38	popPK by multivariate linear regression	AUC0-12-to-adverse effects ratio	Accurate prediction of the target; Black recipients showed higher AUC and Cl; greater adverse effects were found in women (and more in Black women)	MAE%
Vadcharavivad, 2016 [123]	Thailand	Retrospective study	Kidney	N/A	96, -	44.67, - [Mean]		popPK (1CMT with first-order absorption)	CL/F, V/F	Accurate prediction of the target; hemoglobin and duration of TAC therapy could contribute to interindividual variabilities	*p*-value
Valdivieso, 2013 [124]	Spain	Retrospective study	Liver	0–15 days	50, -	N/A	N/A	popPK (compartmental model with first-order conditional estimation method)	Significant predictors	Accurate prediction of the target; low HCT and ALB contribute to TAC concentration	GoF, Bootstrap (1000 runs), VPC
vanBoekel, 2015 [125]	The Netherlands	Retrospective study	Kidney	>6 months	26, -	43.9, - [Median]	69	LSS	AUC0-24	Accurate prediction of the target using C0, C2, and C4	SEE%,
Velickovic-Radovanovic, 2010 [126]	Serbia	Retrospective study	Kidney	N/A	18, -	40.11, - [Mean]	55	Multiple stepwise regression analysis	AUC0-12	Accurate prediction of the target using C1.5, C4, and C8; women show significantly lower AUC values	MPPE, MAPE
Velickovic-Radovanovic, 2015 [127]	Serbia	Prospective study	Kidney	N/A	20, 16	38.2, 40.6 [Mean]	50	popPK (non-compartment)	AUC0-12	Gender-dependent pharmacokinetics in a steady state in terms of best sampling time in which measured Tac concentration best predicts AUC value (accurate prediction of the target using C2 in females and C1, C4 and C12 in males)	R, R2
Wang, 2020 [128]	China	Retrospective study	Kidney	N/A	406, -	32.25, - [Median]	73.89	Regression Tree	Initial dose, significant predictors	CYP3A5 and hemoglobin influence C0/D initial dose	*p*-value, R
Wang, 2022 [129]	China	Retrospective study	Kidney	3–215 days	88 (65 PK, 23 LSS), -	44, - [Mean]	64.6	Non-compartmental PK, BE and LSS	AUC0-12	Accurate prediction of the target using C4, C4, C6 and C10; patients with specific genotypes had higher AUC than the rest	PE, APE, MPE MAPE, R2, GoF
Woillard, 2011 [130]	France	Retrospective study	Kidney	Weeks 1, 2 and months 1, 3, 6 and 12	49, 24	53.78, 53.78 [Mean]	52	popPK (2CMT with Erlang absorption (n = 3) and first-order elimination), and BE	AUC0-12	Accurate prediction of the target by Bayesian estimator	VPC, MPE, RMSE
Woillard, 2017 [131]	France	Retrospective study	Kidney and liver	≥6 months (0.5–14.25 years)	73, 24 Kidney- 85, 28 liver	50 (kidney), -; 52 (liver), -	N/A	popPK (1CMT with first-order elimination and one or two absorption phases described by a sum of two gamma distributions), BE	AUC0-24	Accurate prediction of the target using C0, C1, and C3	VPC
Woillard, 2021 [132]	France	Retrospective study	Kidney, liver, heart, lung, other	-	2126, 709	51.5, 51.5 [Median]	N/A	XGBoost (2 or 3 sampling times)	AUC0-12, AUC0-24	Accurate prediction of the target; XGBoost had superior performance compared with traditional PK modeling with Bayesian estimation	RMSE, R2
Woillard, 2023 [133]	France	Retrospective study	Kidney	<3 and >12 months	1325, -	51, - [Median]	N/A	Pearson correlation or Bonferroni-corrected Tukey post-tests between AUC and C0	C0	AUC/C0 ratio is stable in large populations and can be used to estimate C0 in individuals	R
Yoon, 2022 [134]	Republic of Korea	Retrospective study	Liver	N/A	434, -	N/A	N/A	LSTM and GBM	Initial dose	LSTM had better performance	RMSEMDPE, MDAPE
Zhang, 2022 [135]	China	Retrospective study	Kidney	>3 months	5439, -	32, - [Median]	69.8	GBDT, RF, SVR, KNN, LASSO, RR, LR, and TabNet	Next dose	TabNet algorithm outperformed other algorithms with the highest R2	R2, MAE, MSE, RMSE, and percentage of overestimated/underestimated dose in the testing cohort
Zhang, 2022 [136]	China	Retrospective study	Kidney	≤21 days	240, -	39.4, - [Mean]	73	popPK (2CMT with first-order absorption and elimination)	pk parameters	Accurate prediction of the target in terms of Wuzhi capsule coadministration, 2-CMT model fit data better than 1-CMT model	MAE, MPE, F20%, F30%
Zhao, 2016 [18]	China	Retrospective study	Kidney	3–90 days	-, 52	-, 38.9 [Mean]	67	popPK (1 and 2CMT, steady-state, and Michaelis–Menten), external validation	Next dose, significant predictors	Published models were unsatisfactory in prediction- and simulation-based diagnostics, and thus inappropriate for direct extrapolation correspondingly	PE%, MDPE, MDAE, F20, F30, VPC, IPE%, MIPE%, MAIPE%, IF20, and IF30
Zhu, 2013 [137]	China	Retrospective study	Liver	N/A	26, -	52.57, - [Mean]	84.6	LSS	AUC0-12	Accurate prediction of the target using C0 and C4	PE%, APE%
Zhu, 2022 [138]	China	Retrospective study	Liver	51–150 days	112, 25	49, 50 [Median]	74.45	MLR	Significant predictors associated with C0/D	11 metabolites (including microbiota-derived uremic retention solutes, bile acids, steroid hormones, and medium- and long-chain acylcarnitine) and clinical information found to be suitable predictors	R, ME, MAE, MRE, RMSE

## Data Availability

The original contributions presented in this study are included in the article. Further inquiries can be directed to the corresponding authors.

## Supplementary Materials

The following supporting information can be downloaded at https://www.mdpi.com/article/10.3390/pharmaceutics18040430/s1, Supplementary Material S1 includes PRISMA checklist. Supplementary Material S2 includes the Medline search strategy. Supplementary Material S3 includes Table S1: Critical appraisal for non-randomized control trials, and Table S2: Critical appraisal for randomized control trials. Supplementary Material S4 includes Table S3. Definition of performance metrics used in the literature, Table S4: Model fit metrics, Table S5: Validation metrics, Table S6: Summary statistics, and Table S7. Meta analysis metrics. Supplementary Material S5 includes Table S8. GRADE certainty of evidence ratings. Supplementary Material S6 includes Table S9. CYP3A5 Genotype Effect on CL/F, the Most Dominant Covariate, Table S10. Machine Learning Models, Predictive Performance (Kidney and Liver Transplant). NR indicates that the metric was not reported (not that it was zero or not applicable), Table S11. Population Pharmacokinetic (popPK) Models’ Predictive Performance (Kidney and Liver Transplant). NR indicates that the metric was not reported (not that it was zero or not applicable), Table S12. External Validation Studies of Tacrolimus Population Pharmacokinetic Models. Model-Development Studies with External Validation (*n* = 7), Table S13. External Validation Studies of Tacrolimus Population Pharmacokinetic Models. Standalone External Evaluation Papers (*n* = 5).

## Abbreviations

The following abbreviations are used in this manuscript:

%PRED20	Percentage of measured blood levels predicted within ±20% interval
1CMT	One compartment
2CMT	Two compartments
AdaBoost	Adaptive Boosting
ANN	Artificial neural network
APE%	Absolute prediction error
AST	Aspartate aminotransferase
AUC	Area under concentration curve
BART	Bayesian additive regression tree
BE	Bayesian estimation
Bias	Median percentage predictive error
BRT	Boosted regression tree
CatBoost	Categorical Boosting
F20	Prediction errors within ±20% of the actual values
F30	Prediction errors within ±30% of the actual values
GBDT	Gradient boosted decision tree
GBM	Gradient boosting machine
GMFEs	Geometric mean fold errors
GOF	Goodness-of-fit plots
IF20%	F20 of individual prediction error%
IF30%	F30 of individual prediction error%
Imprecision	Median absolute percentage predictive error
IPE%	Individual prediction error%
KNN	K-nearest neighbor
LASSO	Least Absolute Shrinkage and Selection operator regression
LightGBM	Light Gradient Boosting Machine
LR	Linear regression
LSS	Limited sampling strategy
LSTM	Long short-term memory
MAE	Mean absolute error
MAIPE	Median absolute individual prediction error%
MARS	Multivariate adaptive regression spline
ME	Mean error
MIPE%	Median individual prediction error%
ML	Machine learning
MLP	Multilayer perceptron regression
MLR	Multiple linear regression
MPE	Median prediction error
MRDs	Mean relative deviations
MRE	Mean relative error
MSE	Mean squared error
PBPK	Physiologically based pharmacokinetic
PE	Prediction error
PK	Pharmacokinetics
POD	Post-operative days
popPK	Population pharmacokinetics
RF	Random forest
RL	Reinforcement learning
RMSE	Root mean square error
RMSECV	Root-mean-squared error of cross-validation
RR	Ridge regression
RT	Regression tree
SE	Standard error
SVM	Support vector machine
SVR	Support vector regression
TabNet	Tabular network
TDM	Therapeutic drug monitoring
TTR	Time in therapeutic range
VPC	Visual predictive check
XGBoost	Extreme gradient boosting
