# Supplementary material for: Analytical Models to Optimize Tacrolimus Dosing in Solid Organ Transplantation: A Systematic Review
Source: Pharmaceutics. 2026 Mar 31;18(4):430. doi: 10.3390/pharmaceutics18040430 (PMC13119010; doi:10.3390/pharmaceutics18040430)
Supplement: Supplementary file 1 [file pharmaceutics-18-00430-s001.zip › Supplementary material S2.pdf]

Supplementary Material S2 - Medline search strategy

Ovid MEDLINE(R) ALL <1946 to April 30, 2024>

1 Tacrolimus/ 18183

2 Tacrolimus.tw,kf. 19833

3 (prograf or prograft or FR 900506 or FR900506 or FK-506 or FK506).tw,kf. 8782

4 1 or 2 or 3 29769

5 exp artificial intelligence/ 195294

6 (machine learn* or artificial intelligence).tw,kf. 148842

7 reinforcement learn*.tw,kf. 7070

8 q learn*.tw,kf. 597

9 transfer learn*.tw,kf. 6422

10 deep learn*.tw,kf. 63993

11 (Hierarchical adj2 learn*).tw,kf.654

12 machine intelligence.tw,kf. 336

13 models, statistical/ 100109

14 (predict* adj3 model*).tw,kf. 201221

15 (Policy Iteration or Value Iteration or Markov or Optimal Policy or Policy Evaluation or Monte Carlo or Off-policy Prediction or On-policy Prediction or Temporal Difference Learning or On-Policy TD control or Off-Policy Temporal Difference control or Off-Policy TD control or Sarsa or n-step Bootstrapping or n-step off-policy Learning or Approximation solution method or Bellman Equation or Actor-critic Method or Reinforcement Learning with human Feedback or RLwF or Q-function or Q-network).mp. 100860

16 bayes theorem/ 52613

17 bayesian.tw,kf. 72199

18 bayes theorem.tw,kf. 1305

19 neural network*.tw,kf. 111695

20 Perception/ 44704

21 perception.tw,kf. 235460

22 linear models/ 87701

23 (linear adj (model* or regression)).tw,kf. 195783

24 (supervis* adj (learn* or machine learn*)).tw,kf.6583

25 (semisupervis* adj (learn* or machine learn*)).tw,kf. 227

26 kinetic model*.tw,kf. 24051

27 or/5-26 1251283

28 4 and 27 679

29 exp animals/ not humans/ 5217727

30 ((p?ediatric* or child**) not adult*).ti. 1078747

31 28 not (29 or 30) 568
