# Supplementary material for: Analytical Models to Optimize Tacrolimus Dosing in Solid Organ Transplantation: A Systematic Review
Source: Pharmaceutics. 2026 Mar 31;18(4):430. doi: 10.3390/pharmaceutics18040430 (PMC13119010; doi:10.3390/pharmaceutics18040430)
Supplement: Supplementary file 1 [file pharmaceutics-18-00430-s001.zip › Supplementary material S3.pdf]

Supplementary Material S3 – Critical scoring of reviewed literature using the Joanna Briggs Institute Meta-Analysis of Statistics Assessment and Review

Table S1: Critical appraisal for non-randomized control trials

Study	Was study based on random or pseudo sample?	Were the criteria for inclusion in the sample clearly defined?	Were confounding factors identified and strategies to deal with them stated?	Were outcomes assessed using objective criteria?	If comparisons are being made, was there sufficient description of the groups?	**Was follow up carried out over a sufficient time period?**	**Were the outcomes of people who withdrew described and included in the analysis?**	**Were outcomes measured in a reliable way?**	**Was appropriate statistical analysis used?**	**Total (%)**
Abderahmene, 2024	0	1	1	1	1	1	0	1	1	77.77
Al-Kofahi, 2021	0	1	1	1	1	1	0	1	1	77.77
Andreu, 2015	0	1	1	1	1	1	0	1	1	77.77
Andreu, 2017	0	1	1	1	1	1	0	1	1	77.77
Andrews, 2019	0	1	1	1	1	1	0	1	1	77.77
Antignac, 2005	0	1	1	1	N/A	1	0	1	1	75
Antignac, 2011	0	1	1	1	1	1	0	1	1	77.77
Åsberg, 2013	0	1	1	1	N/A	1	0	1	1	75
Barraclough, 2011	0	1	1	1	N/A	1	0	1	1	75
Barraclough, 2012	0	1	1	1	N/A	1	0	1	1	75
Barraclough, 2022	0	1	1	1	1	1	0	1	1	77.77
BenFredj, 2016	0	0.5	0.5	1	N/A	1	0	1	1	62.5
Ben-Fredj, 2020	0	0.5	1	1	N/A	1	0	1	1	68.75

Ben-Fredj, 2023	0	0.5	0	1	N/A	1	0	1	1	56.25
Benkali, 2009	0	0.5	0.5	1	N/A	1	0	1	1	62.5
Benkali, 2010	1	0.5	0.5	1	N/A	1	0	1	1	75
Birdwell, 2012	0	0.5	0.5	0.5	N/A	1	0	1	1	56.25
Brooks, 2021	0	1	0.5	1	N/A	1	0	1	1	68.75
Cai, 2020 (#176)	0	1	0.5	1	N/A	1	0	1	1	68.75
Cai, 2020	0	1	1	1	N/A	1	0	1	1	75
Cai, 2022	0	0.5	0.5	1	N/A	1	0	1	1	62.5
Catic-Dordevic, 2018	0	0	0	1	N/A	0	0	0.5	0.5	25
Chen, 1999	1	1	1	1	1	1	0	1	1	88.88
Chen, 2005	0	0.5	0.5	1	1	1	0	1	1	66.66
Chen, 2017	0	0.5	1	1	N/A	1	0	1	1	68.75
Chen, 2021	0	1	0.5	1	N/A	1	0	1	1	68.75
Choshi, 2024	0	0.5	0.5	0.5	N/A	0	0	0.5	0.5	31.25
Damon, 2017	1	0.5	1	1	N/A	1	0	1	0.5	75
Dansirikul, 2004	0	0.5	1	1	N/A	1	0	1	1	68.75
Decrocq-Rudler, 2021	1	1	0.5	1	N/A	1	0	1	1	81.25
Du, 2022	1	1	1	1	N/A	1	0	1	1	87.5
Du, 2024	1	1	0.5	1	N/A	1	0	1	1	81.25
Du, 2024 (#4)	0	1	1	1	N/A	1	0	1	1	75
Elens, 2011	0.5	0.5	0.5	0.5	N/A	1	0	1	1	62.5
El-Nahhas, 2022	0.5	1	1	1	N/A	1	0	1	1	81.25
Faelens, 2022	0	0.5	1	1	N/A	1	0	1	1	68.75
Francke, 2022	0	0.5	1	1	N/A	1	0	1	1	68.75
Francke, 2022 (#2788)	0	1	1	1	1	1	0	1	1	77.77
Fu, 2022	1	1	1	1	N/A	1	0	0.5	1	81.25
Gaies, 2013	0	0	0.5	0.5	N/A	1	0.5	0.5	1	50
Gérard, 2014	0	0	0.5	0.5	N/A	1	0.5	1	1	56.25
Grover, 2011	0	0.5	1	1	N/A	1	0.5	0.5	1	68.75
Han, 2014	0	1	1	0.5	N/A	1	0.5	1	1	75

Han, 2019	0	1	1	0.5	N/A	1	0.5	1	1	75
Itohara, 2022	0	0.5	1	0.5	N/A	1	0	1	0.5	56.25
Ji, 2018	0	0.5	1	0.5	N/A	1	0.5	1	0.5	62.5
Jing, 2021	0	1	1	0.5	N/A	1	0.5	1	1	75
Kim, 2012	0	0.5	1	1	N/A	1	0	0.5	0.5	56.25
Kim, 2012 (#384)	0	1	1	1	N/A	1	0	0.5	0.5	62.5
Kim, 2019	0	0.5	0.5	1	N/A	1	0.5	1	1	68.75
Kirubakaran, 2022	0	1	1	1	N/A	1	0.5	1	1	81.25
Kirubakaran, 2023	0	1	1	1	N/A	1	0.5	1	1	81.25
Kirubakaran, 2024	0	1	1	1	N/A	1	0.5	1	1	81.25
Kuypers, 2004	0	1	1	1	N/A	1	0.5	0.5	1	75
Langers, 2008	0	1	1	1	N/A	1	0.5	1	1	81.25
Li, 2007	1	1	1	1	N/A	1	0.5	1	1	93.75
Li, 2011	0	1	1	1	N/A	1	0.5	0.5	0.5	68.75
Li, 2023	1	1	1	1	N/A	1	0.5	1	1	93.75
Ling, 2020	0	1	1	1	N/A	1	0.5	1	1	81.25
Liu, 2016	0	0.5	0.5	1	N/A	1	0.5	1	1	68.75
Liu, 2020	0	0.5	1	1	N/A	1	0.5	0.5	0.5	62.5
Loer, 2023	0	0.5	0.5	1	N/A	1	0.5	1	1	68.75
Macchi-Andanson, 2001	0	0.5	0.5	1	N/A	1	0.5	1	1	68.75
Marquet, 2021	0	1	0.5	1	N/A	1	0.5	1	1	75
Mathew, 2008	0	0.5	0.5	1	N/A	1	0.5	1	1	68.75
Matsuda, 2022	0	1	0.5	1	N/A	1	0.5	0.5	1	68.75
Methaneethorn, 2022	0	1	1	0.5	N/A	1	0.5	1	1	75
Moes, 2016	0	1	1	0.5	N/A	1	0.5	1	1	75
Musuamba, 2009	0	0	0.5	1	N/A	1	0.5	1	1	62.5
Musuamba, 2013	0	0	0.5	0.5	N/A	1	0.5	1	1	56.25
Nanga, 2019	0	0	0	1	N/A	1	0.5	1	1	56.25

Nguyen, 2023	1	1	1	1	0.5	1	0.5	1	1	88.88
Niioka, 2015	0	0.5	0.5	0.5	N/A	1	0.5	1	1	62.5
OpdenBuijsch, 2007	0	0.5	0.5	0.5	N/A	1	0.5	1	1	62.5
Oteo, 2013	0.5	0.5	1	1	1	1	0.5	1	1	83.33
Pankewycz, 2020	0	0.5	1	0.5	1	1	0.5	0.5	0.5	61.11
Pei, 2023	0	0.5	0.5	1	1	1	0.5	1	1	72.22
Ragette, 2005	0	0.5	0.5	1	N/A	1	0.5	1	1	68.75
Resendiz-Galvan, 2019	0	0.5	1	1	1	1	0.5	1	1	77.77
Riff, 2019	1	1	1	1	N/A	1	0.5	1	1	93.75
Rong, 2019	0	0.5	1	1	N/A	1	0.5	1	1	75
Saint-Marcoux, 2005	0	0.5	1	1	N/A	1	0.5	1	0.5	68.75
Saint-Marcoux, 2010	0	0.5	1	0.5	N/A	1	0.5	1	0.5	62.5
Saint-Marcoux, 2011	0.5	0.5	1	1	1	1	0.5	1	1	83.33
Saint-Marcoux, 2013	0	0.5	1	0.5	N/A	1	0.5	1	1	68.75
Sallustio, 2021	0	0.5	1	1	N/A	1	0.5	1	1	75
Scholten, 2005	1	0.5	0.5	1	1	1	0.5	1	1	83.33
Smith, 2023	0	0	0	1	N/A	0.5	0.5	1	1	50
Stefanovic, 2015	0	1	1	1	N/A	1	0.5	0.5	1	75
Stifft, 2020	0	1	0.5	0.5	1	1	0.5	1	1	72.22
Storas, 2022	0	0.5	0.5	0.5	1	1	0.5	1	1	66.66
Storset, 2014	0	0.5	1	1	1	1	0.5	1	1	77.77
Tang, 2017	0	0.5	1	0.5	1	1	0.5	0.5	0.5	61.11
Tornatore, 2022	0	1	0.5	0.5	N/A	1	0.5	0.5	1	62.5
Vadcharavivad, 2016	0	0.5	1	1	N/A	1	0.5	1	1	75
Valdivieso, 2013	0	0.5	0.5	1	N/A	1	0.5	1	0.5	62.5

vanBoekel, 2015	0	0.5	0.5	1	N/A	1	0.5	1	1	68.75
Velickovic-Radovanovic, 2010	0	0.5	1	1	N/A	1	0.5	1	1	75
Velickovic-Radovanovic, 2015	0	0.5	1	1	N/A	1	0.5	1	1	75
Wang, 2020	0	0.5	1	1	N/A	1	0.5	1	0.5	68.75
Wang, 2022	0	0.5	0.5	1	N/A	1	0.5	1	1	68.75
Woillard, 2011	0	0.5	0.5	1	N/A	1	0.5	1	1	68.75
Woillard, 2017	0	0.5	1	1	N/A	1	0.5	1	1	75
Woillard, 2021	0	0.5	0.5	1	1	1	0.5	1	1	72.22
Woillard, 2023	0	0.5	0.5	1	1	1	0.5	1	1	72.22
Yoon 2022	0	0	0	1	0.5	1	0.5	1	1	55.55
Zhang, 2022	0	1	1	1	N/A	1	0.5	1	1	81.25
Zhang, 2022 #101	0	0.5	1	1	N/A	1	0.5	1	1	75
Zhao, 2016	0	0.5	1	1	1	1	0.5	1	1	77.77
Zhu, 2013	0	0.5	1	1	N/A	1	0.5	1	1	75
Zhu, 2022	0	1	1	1	N/A	1	0.5	1	1	81.25

Table S2: Critical appraisal for randomized control trials

Study	Was true randomization used for assignment of participants to treatment groups?	Was allocation to treatment groups concealed?	Were treatment groups similar at the baseline?	Were participants blind to treatment assignment?	Were those delivering the treatment blind to treatment assignment?	Were treatment groups treated identically other than the intervention of interest?	Were outcome assessors blind to treatment assignment?	Were outcomes measured in the same way for treatment groups?	Were outcomes measured in a reliable way?	Was follow up complete and if not, were differences between groups in terms of their follow up adequately described and analysed?	Were participants analyzed in the groups to which they were randomized?	Was appropriate statistical analysis used?	Was the trial design appropriate and any deviations from the standard RCT design (individual randomization, parallel groups) accounted for in the conduct and analysis of the trial?	Total (%)
Allard, 2019	1	0.5	1	0	0	1	1	1	1	0.5	1	1	0.5	73.07
Lloberas, 2023	1	0.5	1	0	0	1	0	1	1	1	1	1	0.5	69.23
Marquet, 2018	1	0.5	1	0	0	1	0	1	1	0.5	1	1	0.5	65.38

Shi, 2023	1	0.5	1	0	0	1	0	1	1	0.5	1	1	0.5	65.38
