# Supplementary material for: Analytical Models to Optimize Tacrolimus Dosing in Solid Organ Transplantation: A Systematic Review
Source: Pharmaceutics. 2026 Mar 31;18(4):430. doi: 10.3390/pharmaceutics18040430 (PMC13119010; doi:10.3390/pharmaceutics18040430)
Supplement: Supplementary file 1 [file pharmaceutics-18-00430-s001.zip › Supplementary material S4.pdf]

Supplementary Material S4 – Quality metrics and tools

Table S3. Definition of performance metrics used in the literature

Metric	Formula	Description
Prediction error (PE)	$\mathrm{PE} = \mathrm{PRED} - \mathrm{OBS}$	Difference between predicted and observed concentrations
Percent Error (PE%)	PE% = (PRED-OBS)/OBS *100	Percentage difference between predicted and observed concentrations
Mean Prediction Error (MPE)	MPE = $\frac{\sum(\mathrm{PRED}-\mathrm{OBS})}{\mathrm{n}}$	Average of prediction errors; measures systematic bias
Median Prediction Error (MDPE)	MDPE = median(PRED - OBS)	Median of prediction errors; robust measure of central tendency
Median Percentage Error (MDPE%)	MDPE% = median(PE%)	Median of percentage errors; robust measure of systematic bias
Mean Absolute Error (MAE)	MAE = (1/n) × Σ|PRED - OBS|	Average of absolute prediction errors; measures precision
Mean Absolute Percentage Error (MAPE%)	MAPE% = $\frac{\sum\lvert PE\%\rvert}{\mathrm{n}}$	Average of absolute percentage errors; measures precision
Median Absolute Prediction Error (MDAPE)	MDAPE = median(|PRED - OBS|)	Median of absolute prediction errors; robust measure of precision
Median Absolute Percentage Error (MAPE%)	MAPE% = median(|PE%|)	Median of absolute percentage errors; robust measure of precision
root mean squared error (RMSE)	$\mathrm{RMSE} = \sqrt{\frac{1}{\mathrm{n}}\sum_{\mathrm{n}=1}^{\mathrm{n}}(\mathrm{PRED}-\mathrm{OBS})^2}$	Square root of the average squared prediction errors; penalizes large errors
Root Median Squared Error (RMSE)	RMSE = $\sqrt{[\mathrm{median}((\mathrm{PRED}-\mathrm{OBS})^2)]}$	Square root of the median squared prediction errors; robust against outliers
Relative Root Mean Squared Error (rRMSE)	rRMSE = RMSE/mean(OBS)	RMSE normalized by the mean observed concentration
F_{20}	Percentage of PE% within ±20%	Proportion of predictions within 20% of observed values
F_{30}	Percentage of PE% within ±30%	Proportion of predictions within 30% of observed values

Table S4: Model fit metrics

Metric	Formula	Description
Coefficient of determination (R^2)	$R^2 = 1 - [\Sigma(\mathrm{OBS} - \mathrm{PRED})^2]/[\Sigma(\mathrm{OBS} - \mathrm{mean(OBS)})^2]$	Proportion of variance in observations explained by the model
Correlation coefficient (R)	$R = \Sigma[(\mathrm{OBS} - \mathrm{mean(OBS)})(\mathrm{PRED} - \mathrm{mean(PRED)})]/[\surd(\Sigma(\mathrm{OBS} - \mathrm{mean(OBS)})^2 \times \Sigma(\mathrm{PRED} - \mathrm{mean(PRED)})^2)]$	Measure of linear correlation between observed and predicted values
Goodness-of-fit plots (GOF)	Visual comparison of observed vs. predicted values	Graphical assessment of model fit quality
Bland-Altman plots	Plot of difference (PRED - OBS) vs. average ((PRED + OBS)/2)	Visual assessment of agreement between two methods

Table S5: Validation metrics

Metric	Description
Visual Predictive Check (VPC)	Graphical comparison of observed concentrations against prediction intervals generated from model simulations (typically 1,000 simulations)
Prediction-corrected VPC (pcVPC)	VPC that normalizes for differences in dose and covariate values across the population
Bootstrap	Resampling technique (typically 1,000 runs) that evaluates parameter uncertainty and stability
Jackknife validation	Leave-one-out cross-validation technique to assess model stability
Normalized Prediction Distribution Errors (NPDE)	Statistical metric that evaluates if model predictions follow the same distribution as observations

Table S6: Summary statistics

Metric	Description
Akaike Information Criterion (AIC)	Statistical measure of model quality that penalizes complexity: AIC $= -2\ln(L) + 2k$

Bayesian Information Criterion (BIC)	Statistical measure similar to AIC with stronger penalty for complexity: BIC = $-2\ln(L) + k \cdot \ln(n)$
Objective Function Value (OFV)	Value of the objective function being minimized during model development; lower values indicate better fit

PRED = Model-predictions

OBS = Observed values

n = Number of observations

L = Maximum likelihood

k = Number of model parameters

Σ = Sum operator

Table S7: Meta analysis metrics

Metric	Formula	Definition
Odds Ratio (OR)	$OR = \frac{Odds\ of\ event\ in\ the\ experiment\ group}{Odds\ of\ event\ in\ the\ control\ group}$	a measure to evaluate the exposure to a treatment in experimental group and an outcome compared to the control group.
