# Supplementary material for: Analytical Models to Optimize Tacrolimus Dosing in Solid Organ Transplantation: A Systematic Review
Source: Pharmaceutics. 2026 Mar 31;18(4):430. doi: 10.3390/pharmaceutics18040430 (PMC13119010; doi:10.3390/pharmaceutics18040430)
Supplement: Supplementary file 1 [file pharmaceutics-18-00430-s001.zip › Supplementary material S5.pdf]

Supplementary Material S5 – Grading of Recommendations Assessment, Development and Evaluation (GRADE)of Evidence Rating

Table S8. GRADE certainty of evidence ratings

Outcome domain/ studies	**Sub-outcomes**	**Risk of bias**	**Inconsistency**	**Indirectness**	**Imprecision**	**Publication bias**	**Level of certainty of evidence**
Tacrolimus dosing prediction *[Abderahmene, 2024, Allard, 2019, Andreu, 2015, Andrews, 2019, BenFredj, 2016, Benkali, 2010, Cai, 2020, Cai, 2022, Cai, 2020 (#176), Damon, 2017, Decrocq-Rudler, 2021, Du, 2024, Du, 2022, Fu, 2022, Gérard, 2014, Kim, 2019, Liu, 2020, Niioka, 2015, Shi, 2023, Tang, 2017, Velickovic-Radovanovic, 2010, Zhang, 2022]*	a. PIK parameter prediction<br>b. Proportion of patients with in target ranges<br>c. Outcome efficacy measurement	No serious concerns	No serious concerns	No serious concerns	Minor concerns (some studies rely heavily on physiological assumptions)	No serious concerns	High certainty - ⊕⊕⊕⊕ (minor concerns about imprecision)
Significant influencing factors prediction *[Elens, 2011, Francke, 2022, Kim, 2012, Kuypers, 2004, Li, 2011, Stefanovic, 2015, Tornatore, 2022, Wang, 2020, Zhang, 2022 (#101), Zhu, 2022, Chen, 2021, Jing, 2021]*	a. AUC prediction<br>b. Dose prediction	No serious concerns	No serious concerns	No serious concerns	No serious concerns	No serious concerns	High certainty - ⊕⊕⊕⊕

AUC prediction *[Brooks, 2021, Langers, 2008, Liu, 2016, Mathew, 2008, Musuamba, 2013, Ragette, 2005, Velickovic-Radovanovic, 2015, Zhu, 2013, Saint-Marcoux, 2010, Stifft, 2020, Storas, 2022, Gaies, 2013, Scholten, 2005, vanBoekel, 2015, Barraclough, 2012, Barraclough, 2011, Marquet, 2021, Nguyen, 2023, Smith, 2023, Catic-Dordevic, 2018, Dansirikul, 2004, El-Nahhas, 2022, Pei, 2023, Benkali, 2009, Woillard, 2023, Woillard, 2021, Chen, 2017, Pankewycz, 2020, Saint-Marcoux, 2013]*		a. PK parameter prediction<br>b. Best concentration sampling time identification<br>c. Identification of significant factors affecting clearance rate<br>d. Dose/Blood level prediction	Moderate concerns (some studies show gender imbalance)	No serious concerns	No serious concerns	Minor concerns (lack of confidence intervals in some results, lack of generalizability)	No serious concerns	Moderate certainty - ⨁⨁⨁O (moderate concern with risk of bias and minor concern with imprecision)
Tacrolimus blood level prediction *[Yoon, 2022, Åsberg, 2013, Chen, 1999, Choshi, 2024, Du, 2024 (#8), Saint-Marcoux, 2011, Antignac, 2011, Lloberas, 2023, Ben-Fredj, 2023, Ben-Fredj, 2020, Faelens, 2022,*		a. Identification of significant factors affecting blood	No serious concerns	No serious concerns	No serious concerns	Minor concerns (most studies targeted PK endpoints rather than clinical outcomes)	No serious concerns	High certainty - ⨁⨁⨁⨁ (minor concern with imprecision)

Francke, 2022]	levels<br>b. Influence of the analytical method by modeling the TAC blood concentrations using different assay methods<br>c. Proportion of patients with tacrolimus concentration within target range<br>d. Time to achieve targ						

	et and distance from target						
PK parameter prediction *[Al-Kofahi, 2021, Andreu, 2017, Antignac, 2005, Barraclough, 2022, Birdwell, 2012, Chen, 2005, Grover, 2011, Han, 2014, Han, 2019, Itohara, 2022, Ji, 2018, Kim, 2012 (#1264), Kirubakaran, 2023, Li, 2007, Ling, 2020, Li, 2023, Loer, 2023, Macchi-Andanson, 2001, Methaneethorn, 2022, Moes, 2016, Musuamba, 2009, Oteo, 2013, Resendiz-Galvan, 2019, Riff, 2019, Rong, 2019, Saint-Marcoux, 2005, Storset, 2014]*	a. Identification of significant factors affecting PK<br>b. Dose/Blood level prediction<br>c. AUC prediction	Moderate concerns (most studies targeted specific population or limited time intervals limiting generalizability)	No serious concerns	No serious concerns	Moderate concerns (some studies had limited samples limiting generalizability)	No serious concerns	Low certainty - ⨁⨁OO (moderate concerns with both risk of bias and imprecision)
External validation *[Kirubakaran, 2022, Kirubakaran, 2024, Nanga, 2019, OpdenBuijsch, 2007, Zhao, 2016]*	a. Model predictive evaluation<br>b. Influencing factor prediction	No serious concerns	No serious concerns	No serious concerns	No serious concerns	No serious concerns	High certainty - ⨁⨁⨁⨁
	c.						

⨁⨁⨁⨁ - high certainty, ⨁⨁⨁O - moderate certainty, ⨁⨁OO - low certainty, ⨁OOO - very low certainty.
