# Supplementary material for: Analytical Models to Optimize Tacrolimus Dosing in Solid Organ Transplantation: A Systematic Review
Source: Pharmaceutics. 2026 Mar 31;18(4):430. doi: 10.3390/pharmaceutics18040430 (PMC13119010; doi:10.3390/pharmaceutics18040430)
Supplement: Supplementary file 1 [file pharmaceutics-18-00430-s001.zip › Supplementary material S6.pdf]

Supplementary Material S6 – supplementary tables

Table S9. CYP3A5 Genotype Effect on CL/F, The Most Dominant Covariate

Study (Year)	Organ	CYP3A5 Effect on CL/F	Notes
**Al-Kofahi et al. (2021)**	Kidney	*1/*3: +181%; *1/*1: **+305%** vs *3/*3	European ancestry; largest reported effect
**Andrews et al. (2019)**	Kidney	*1/*3 or *1/*1 combined: **+62%** vs *3/*3	Shared parameter for both heterozygous and homozygous
**Moes et al. (2016)**	Liver	*1/*3: **+38%** vs *3/*3	Multiple donor/recipient genotype combinations up to +71%
**Reséndiz-Galván et al. (2019)**	Kidney	*1/*1: **+111%**; *1/*3: **+53%** vs *3/*3	Mexican population; CL = 26, 18.8, 12.3 L/h respectively
**Abderahmene et al. (2024)**	Kidney	Expressers: **+26%** vs non-expressers	Tunisian population; weaker effect likely due to low *1 frequency
**Åsberg et al. (2013)**	Kidney	*1/*3: **+26%** vs *3/*3	Nonparametric model

Table S10. Machine Learning Models, Predictive Performance (Kidney and Liver Transplant). NR indicates the metric was not reported (not that it was zero or not applicable).

Study (Year)	Organ	ML Method	Validation Type	n (val)	F20(%)-F230(%)	RMSE / rRMSE	MAPE (%)	R^2 / r	Bias / MPE	Head-to-head vs. popPK
**Kidney Transplant**										
**Fu et al. (2022)**	Kidney	Extra Trees Regression	Internal test set	511	92.8-NR	NR	NR	0.85	NR	Not directly reported
**Cai N. et al. (2020)**	Kidney	Random Forest (CYP3A4/5-integrated)	Cross-validation	114	NR	NR	NR	0.92–0.95	NR	Not reported
**Storas et al. (2022)**	Kidney	XGBoost (AUC via LSS)	External (multi-centre)	68–740	NR	9.7–9.9% (rRMSE)	NR	NR	NR	Yes. mixed; ML better for flexible time points
**Woillard et al. (2021)[a]**	Kidney / Liver / Heart	XGBoost (AUC via LSS)	External (multi-centre)	Multiple	NR	5.0–8.6% (rRMSE, b.i.d.)	NR	0.94–0.98	0.5–1.1%	Yes. ML superior to MAP-BE in most datasets
**Faelens et al. (2022)**	Kidney	MIPD / Bayesian feedback	Prospective simulation	200 (sim)	NR	0.361 ng/mL (abs)	NR	NR	NR	Yes. MIPD: PTA 39% vs. 28% standard
**Liver Transplant**										
**Woillard et al. (2021)[a]**	Liver	XGBoost (AUC via LSS)	External	Multiple	NR	See kidney[a]	NR	0.91–0.95	≤1%	Yes. ML superior to MAP-BE in most datasets
**Du et al. (2023)**	Liver	Artificial Neural Network (ANN)	Internal test set	57 samples	NR	NR	NR	0.97 (r)	0.27 ± 0.75 ng/mL (ME)	Not reported

**Shi et al. (2023)**	Liver	Genotype-guided logistic model	Prospective pilot trial	40	NR	NR	NR	AUC-ROC 0.79 (val)	NR	vs. clinician-based dosing; fewer dose adjustments in ML arm
**Li et al. (2023) [stable]**	Liver	Limited Sampling Model (LSM)	Internal	23 patients	NR	NR	17 (C4/C6)	0.97–0.98	0.7%	Not reported; stable phase only

[a] Woillard et al. (2021) covers kidney, liver, and heart transplant recipients within a single multi-organ study; performance metrics reported per formulation (b.i.d./q.d.) and sampling scenario (2 or 3 concentrations). The rRMSE of 5.0% corresponds to the best scenario (b.i.d., 3 samples, training set). External validation rRMSE values were higher in some cohorts.

Table S11. Population Pharmacokinetic (popPK) Models' Predictive Performance (Kidney and Liver Transplant). NR indicates the metric was not reported (not that it was zero or not applicable).

Study (Year)	Organ	Software / Approach	Validation Type	n (val)	F20 (%)-F230(%)	RMSE / rRMSE	MAPE (%)	R^2 / r	Bias / MPE	Phase / Notes
**Kidney Transplant — Model-Development Papers**										
**Al-Kofahi et al. (2021)**	Kidney	NONMEM (FOCE-I)	External (DeKAF Genomics)	1,361	NR	1.73 ng/mL (indiv.)	6.5	NR	ME: 0.49 ng/mL	Early + stable; largest validation cohort
**Andrews et al. (2019)**	Kidney	NONMEM	External hold-out	304	NR	NR	NR	NR	Acceptable	First 3 months post-Tx
**Ben-Fredj et al. (2023)**	Kidney	NONMEM	Prospective (external)	30	NR	NR	2.1	NR	NR	Prospective; early + stable
**Storset et al. (2014)**	Kidney	NONMEM (theory-based)	External (independent cohort)	72	NR	NR	NR	NR	MPE –1.2%	Early phase (14 days); superior to empirical
**Åsberg et al. (2013)**	Kidney	NPAG (nonparametric)	Internal (LOO-CV)	~150	NR	NR	NR	0.86 (indiv.) 0.33 (pop.)	NR	Stable; CYP3A5-stratified
**Kidney Transplant — Multi-Model Evaluation Papers**										

**Methaneethorn et al. (2022) — 10 models**	Kidney (Thai)	Various	External (prospective cohort)	75	NR	NR	3/10 models <50%; 8/10 unsatisfactory	NR	NR	Stable; highlights ethnic transferability gap
**Zhao et al. (2016) — multiple models**	Kidney (Chinese)	Various	External (52 patients, 609 troughs)	52	Variable	NR	Variable	NR	Variable	Stable; MDPE/MAPE varied widely across models
**Kirubakaran et al. (2022) — 17 models**	Heart	Various	External (retrospective)	100	All fail	NR	NR	NR	NR	All 17 models underpredicted with azoles; heart unmet need
**Liver Transplant — Model-Development Papers**										
**Riff et al. (2019)**	Liver	NONMEM + NPAG	Internal (VPC only)	—	NR	NR	NR	NR	NR	Separate early (D7) + stable (W6) models; no external validation
**Moes et al. (2016)**	Liver	NONMEM	Internal (pcVPC only)	—	NR	NR	NR	NR	NR	Stable (>3 months); CYP3A5 + donor genotype
**Antignac et al. (2005)**	Liver	NONMEM	Internal (bootstrap)	—	NR	NR	NR	NR	NR	Early phase; sigmoid CL model
**Liver Transplant — Multi-Model Evaluation Papers[b]**										
**Cai et al. (2020) — 16 models**	Liver	Various	External (84 patients, 572 troughs)	84	NR	NR	NR	NR	6/16 models: MDPE ≤ ±20%	Stable; all NPDEs rejected ($p < 0.01$); global misspecification

For liver transplant, external validation of popPK models is largely absent (Cai 2020 found only 6/16 models meeting MDPE ≤±20%), and ML liver models are evaluated on very small test sets (Du 2023: 57 samples from 23 patients). Neither model class has demonstrated robust external validation for liver transplant.

Table S12. External Validation Studies of Tacrolimus Population Pharmacokinetic Models. Model-Development Studies with External Validation (n = 7)

Study	Transplant	Development cohort	External validation cohort	Validation design	Key covariates	Performance metrics	Notable findings
**Andrews et al., 2019**	Kidney	n = 337 Erasmus MC, Rotterdam, Netherlands First 3 months post-Tx	n = 304, First 3 months post-Tx	Retrospective independent cohort (hold-out)	CYP3A5 (*1/*1, *1/*3, *3/*3); CYP3A4*22; BSA; age; haematocrit; albumin; creatinine	pcVPC: adequate fit MAPE/RMSE/F20/F30: not explicitly reported Starting-dose model validated separately	Explains ~30% of CL/F variability; limited to first 3 months; CYP3A4*22 absent in non-European cohorts
**Al-Kofahi et al., 2021**	Kidney	n = 608 (GEN03 study, 10,992 troughs) Multi-centre USA (U. Minnesota, Mayo Clinic, U. Iowa, U. Manitoba, U. Alberta) 2012–2016	n = 1,361 (DeKAF Genomics, 23,658 troughs) Same multi-centre USA consortium 2005–2010	Retrospective multi-centre independent cohort Largest validation dataset in the development literature	CYP3A5 (*1/*1/*3/*3); CYP3A4*22; weight; age; haematocrit; diabetes; corticosteroid use; CCB use; time post-Tx	ME: 0.49 ng/mL MPE: 6.5% RMSE: 3.09 ng/mL (pop), 1.73 ng/mL (indiv) F20/F30: not reported pcVPC: acceptable	CYP3A5 expressers: +181% (*1/*3) to +305% (*1/*1) on CL/F vs *3/*3; best published individual RMSE; CL/F 18% lower after day 8
**Storset et al., 2014**	Kidney	n = 242 (3,100 samples, combined Australian + Norwegian cohorts) Princess Alexandra Hosp (Brisbane, AU) + Oslo Univ Hospital (NO)	n = 72 (837 samples) Oslo University Hospital, Norway First 3 weeks post-Tx	Retrospective independent hold-out cohort Mechanistic (theory-based) model vs two empirical comparators	CYP3A5; prednisolone dose (time-varying CYP3A4 induction); fat-free mass; BSA; haematocrit; albumin; AST; time post-Tx	MPE (theory-based): −1.2% (95% CI −3.0, +0.1) MPE (empirical models): +1.3% to +1.6% F20/F30/RMSE: not reported pcVPC: adequate for theory-based model	Theory-based model superior to empirical models in early phase (first 14 days); mechanistic prednisone induction function key advantage; after day 14, all models equivalent
**Ben-Fredj et al., 2023**	Kidney	Based on Al-Kofahi 2021 (GEN03, n = 608) Multi-centre USA	n = 30 (279 troughs) Monastir Hospital, Tunisia Prospective, Dec 2016–Sep 2020	Prospective independent cohort	CYP3A5; CYP3A4*22; age; weight; time post-Tx (applied from Al-Kofahi model)	MAPE: 2.1–2.5 ng/mL r^2: 0.746–0.748 92.4% predictions within ±1.96 SD (Bland-Altman) F20/F30/RMSE: not reported	Model-guided dosing: 63.9% in target range vs 38.0% (control); prospective design; small n (single centre); Tunisian population CYP3A5 effect attenuated vs European
**Abderahmene et al., 2024**	Kidney	n = 196 (1,901 samples, development of Tunisian-adapted model) Sahloul University Hospital + Monastir Hospital, Tunisia Jan 2009–Nov 2019	n = 196 (same cohort, hold-out split) Tunisia Retrospective	Retrospective hold-out; Tunisian-adapted version of Dutch (Andrews 2019) model	CYP3A5 (weaker effect vs Europeans); CYP3A4*22 (absent in Tunisian cohort); BSA; age; haematocrit; albumin	MAPE: 2.1–2.5 ng/mL r^2: 0.746 VPC: adequate F20/F30: not reported Model-based dosing: 21.9% supratherapeutic vs 54% with weight-based dosing	CYP3A4*22 irrelevant in Tunisian population (near-zero frequency); CYP3A5 effect attenuated vs European models; ethnic re-calibration required; supratherapeutic exposures reduced ~3-fold

**Faelens et al., 2022**	Kidney	n = 315 de novo kidney recipients (Vanhove 2004–2014 dataset) University Hospital Leuven, Belgium Days 0–14 post-Tx	n = 315 same cohort In silico prospective clinical trial simulation Belgium	In silico MIPD prospective simulation (Bayesian feedback MIPD vs standard physician dosing)	Age; weight; haematocrit; formulation type; lag time; time-dependent CL (CYP3A5 not available in this cohort)	RMSE: 0.361 (base) → 0.307 (full model) PTA: 39% ±15.8% (MIPD) vs 28% ±16.1% (standard care) Log²-distance to target: 0.055 vs 0.080 F20/F30 not reported	MIPD superior to physician dosing from day 5 onward ($p < 0.01$); limited to first 14 days; CYP3A5 genotype unavailable; in silico design; proposes prospective trial of n=200
**Nanga et al., 2019**	Multi-organ (kidney, liver, lung, heart)	76 published popPK models (meta-analysis) Global (multi-centre literature-based) Inception–June 2018	3 independent cohorts Mixed (pediatric + adult; multi-organ) Retrospective from published literature	Retrospective meta-analysis; internal + external validation on literature-derived cohorts	Body weight (allometric: CL exponent 0.75, V exponent 1.0); organ type; time post-Tx; age; fat-free mass	pcVPC: acceptable fit across organ types Systematic bias/RMSE/MAPE: not uniformly reported Pop CL: 22.5 L/h; V_c: 109.7 L	First meta-model spanning kidney, liver, lung, heart; organ type and time post-Tx drive CL/F; CYP3A5/4 polymorphisms not captured; no individual-level genetic data

Table S13. External Validation Studies of Tacrolimus Population Pharmacokinetic Models. Standalone External Evaluation Papers (n = 5)

Study	Transplant	Validation cohort	Models evaluated (n)	Performance range	Best / worst models	Key findings
**Zhao et al., 2016**	Kidney	n = 52 patients 609 trough samples Huashan Hospital, Shanghai, China May 2009–Dec 2013	16 published popPK models	MAPE: 16.2–58.4% MDPE: −40.1% to +13.9% NPDE: all models rejected ($p < 0.01$) Bayesian (2–3 priors): F30 improves >50%	Best: Passey 2011; Storset 2014 (MDPE <±20%, F20 >50%, F30 >60%) Worst: Zuo 2013; Han 2013; Golubovic 2014 (MAPE >50%)	Bayesian forecasting substantially improved performance with 2–3 prior troughs; Michaelis-Menten kinetics superior to linear for Chinese population; model rankings in external evaluation differed from published internal validation statistics
**Cai et al., 2020**	Liver	n = 84 patients 572 trough samples Huashan Hospital, Shanghai, China Jun 2018–Jan 2019	16 published popPK models	MAPE: 16.6–27.4% (best models) MDPE: variable by model NPDE: ALL models failed ($p < 0.01$) F20/F30: <50% for all models at population level Bayesian (2–4 priors): F20/F30 improved >50%	Best: Zhang 2012; Zhu 2014/2015 (F20 ≥35%, F30 ≥50%) Worst: 3 models with MAPE >100%	Only 6/16 models met MDPE ≤±20%; all NPDEs rejected indicating global model misspecification; Bayesian priors substantially improved individual predictions; liver-specific models not obviously superior to kidney-derived models
**Methaneethorn et al., 2022**	Kidney (Thai population)	n = 75 patients 272 trough samples Bhumibol Adulyadej	10 published popPK models	MAPE: 16.6–27.4% (best models) Population level: only 3/10 models achieved acceptable MAPE <50% 8/10	Best: Models 1, 2, 10 (population-level); Models 3, 5, 8 (individual-level MAPE <25%) Worst: Models	8/10 models unsatisfactory for individual dose adjustment at population level; ethnic transferability gap highlighted; haematocrit and CYP3A5 genotype most influential covariates in Thai population; Bayesian estimation rescued most models

		Hospital, Thailand Retrospective		showed satisfactory individual predictions with Bayesian estimation F20/F30: variable	4, 9 (MAPE >50% or >100%)	
**Kirubakaran et al., 2022**	Heart	n = 85 patients 2,387 samples (1,770 with azoles; 617 without) St. Vincent's Hospital, Sydney, Australia Jan–Dec 2017	17 published popPK models	Bias: −88% to +88% (most models underpredicted) Imprecision: 17.5–67.6% IPE <±20%: <55% for all models NO model achieved ±20% bias AND ≤20% imprecision simultaneously	Best (clinically acceptable bias): M2, M7, M15, M16 (95% CI of bias crosses zero) Worst: M3, M5, M8–M10, M13–M14, M17	ALL 17 models demonstrated unacceptable performance; concomitant azole antifungal therapy major confounder (CYP3A inhibition substantially reduces CL/F); no heart-specific validated model exists; models from kidney/liver do not extrapolate to heart transplant
**Kirubakaran et al., 2024**	Lung	n = 43 patients 1,021 samples (548 with azoles; 473 without) St. Vincent's Hospital, Sydney, Australia Jan–Dec 2017	17 published popPK models	Bias: 88% of models underpredicted (range −60% to −20%) Imprecision: 17.5–67.6% IPE <±20%: <50% for all models pcVPC: ALL models inadequate	Best (95% CI bias crosses zero): M2, M7, M15, M16 (same 4 as heart study) Worst: M3–M5, M8–M14, M17	ALL 17 models failed; azole therapy and cystic fibrosis (~16% of cohort) further confound PK; lung-specific model urgently needed; models from kidney/liver/heart do not generalise to lung transplant

Abbreviations: BSA = body surface area; CCB = calcium-channel blocker; CL/F = apparent clearance; CYP3A5 = cytochrome P450 3A5; F20/F30 = fraction of predictions within ±20%/±30% of observed value; FFM = fat-free mass; Hct = haematocrit; MAPE = mean absolute percentage error; MDPE = median percentage prediction error; ME = mean error; MIPD = model-informed precision dosing; MPE = mean percentage error; NPDE = normalised prediction distribution error; pcVPC = prediction-corrected visual predictive check; PK = pharmacokinetics; popPK = population pharmacokinetics; POD = post-operative day; PTA = probability of target attainment; r^2 = coefficient of determination; RMSE = root mean square error; TDM = therapeutic drug monitoring.

NR = not reported.
